# Thermochemical process and compact apparatus for concentrating oxygen in extraterrestrial atmospheres: a feasibility study

**DOI:** 10.1038/s41598-023-31120-x

**Published:** 2023-03-29

**Authors:** Asmaa Eltayeb, Lena Klaas, Leonhard Kölz, Josua Vieten, Martin Roeb, Christian Sattler

**Affiliations:** 1 Institute of Future Fuels, Deutsches Zentrum Für Luft- Und Raumfahrt/German Aerospace Center (DLR) Linder Höhe, 51147 Cologne, Germany; 2 ExoMatter GmbH, Leutstettener Str. 67, 81477 Munich, Germany; 3 Faculty of Mechanical Engineering, Chair for Solar Fuel Production, RWTH Aachen University, 52062 Aachen, Germany

**Keywords:** Astronomy and planetary science, Chemistry, Energy science and technology, Engineering, Materials science, Mathematics and computing, Physics

## Abstract

The Martian atmosphere contains 0.16% oxygen, which is an example of an in-situ resource that can be used as precursor or oxidant for propellants, for life support systems and potentially for scientific experiments. Thus, the present work is related to the invention of a process to concentrate oxygen in the oxygen-deficient extraterrestrial atmosphere by means of a thermochemical process and the determination of a suitable best-case apparatus design to carry out the process. The perovskite oxygen pumping (POP) system uses the underlying chemical process, which is based on the temperature-dependent chemical potential of oxygen on multivalent metal oxide, to release and absorb oxygen in response to temperature swings. The primary goal of this work is therefore to identify suitable materials for the oxygen pumping system and to optimize the oxidation–reduction temperature and time, required to operate the system, to produce 2.25 kg of oxygen per hour under the Martian most-extreme environmental conditions and based on the thermochemical process concept. Radioactive materials such as ^244^Cm, ^238^Pu and ^90^Sr are analyzed as a heating source for the operation of the POP system, and critical aspects of the technology as well as weaknesses and uncertainties related to the operational concept are identified.

## Introduction

The generation of oxygen outside the Earth's atmosphere is a crucial factor for future manned space missions. The return of astronauts to Earth requires large amounts of propellant and usually oxygen as an oxidant for corresponding rocket engines. In addition, oxygen is needed for life support on manned missions and possibly for scientific experiments.

It is anticipated that the first manned space mission outside the Earth system will aim for planet Mars, and probably will be launched in the 20–30 s of the twenty-first century. Corresponding missions are planned by NASA and SpaceX, among others. Both organizations plan to produce oxygen on site on Mars in an unmanned mission that precedes the manned mission. The production of oxygen on Mars (in-situ resource utilization (ISRU)) may also be needed for the unmanned return of samples from the planet Mars to Earth (Mars Sample Return)^1^.

SpaceX plans to produce hydrogen and oxygen on Mars by mining water ice and electrolyzing the water with electricity generated from photovoltaic solar energy. The hydrogen will be converted to methane using carbon dioxide from the Martian atmosphere, while the oxygen will be stored as an oxidant for the return flight^2^. The US space agency NASA plans to produce oxygen on Mars by high-temperature electrolysis of CO_2_ from the Martian atmosphere. In this process, CO_2_ is split into O_2_ and CO. This process is currently being tested aboard the Mars rover Perseverance as part of the MOXIE experiment^3^. On April 20, 2021, it succeeded for the first time in extracting 5.37 g of oxygen from the Martian atmosphere within one hour^4^. For the planned manned mission, NASA estimated the necessity to produce 22.7 tons of liquid oxygen in 420 earth days, which corresponds to an average oxygen production of 2.25 kg per hour^5^. In the publication “Mars Design Reference Architecture 5.0”, it is assumed that an ISRU system for extracting CO_2_ from the Martian atmosphere and generating gaseous oxygen, basically an upscaled MOXIE system, would weigh about one metric ton^6^. Such an upscaled device, as well as the associated power generating equipment, will be used as a reference marker in this study, as our goal is to surpass the current state of the art technology in extraterrestrial oxygen production.

Both NASA and SpaceX plan the use of electrolysis. The electrical energy needed has to be generated from a primary energy source, which involves inherent losses as well as elaborate and heavy technical equipment. As an alternative, we consider thermochemical processes to gather oxygen. Thermochemical processes have been developed at the German Aerospace Center (DLR) in the Institutes of Solar Research and Future Fuels for the production of nitrogen through air separation and the removal of oxygen by splitting water and CO_2_, as well as the storage of oxygen in oxygen pumps^7–14^. These systems can also be used for the concentration of oxygen^15^ and offer advantages over electrolysis from a thermodynamic point of view, since the generation of the necessary concentration gradient is much less energy-intensive than CO_2_ splitting.

This is made possible because the Martian atmosphere contains 0.16% oxygen, resulting in an average oxygen partial pressure of about 1.36 Pa at the surface^16^. Local atmospheric conditions vary considerably over the seasons on Mars and due to local altitude and latitude. This is discussed in more detail in the section on conditions in the Martian atmosphere below.

The basic principle of a perovskite oxygen pump (POP) is the oxidation of a partially reduced perovskite with atmospheric oxygen and the subsequent release of this pure oxygen in an enclosed volume at higher partial pressure by increasing the temperature. Heat from the natural radioactive decay of radioisotopic compounds (RIC) is envisioned as the primary heating source for the perovskite material of this novel POP system. The idea of using RIC comes from long-used nuclear processes to generate heat and electricity for energy needs^17^. Although this method is associated with large-scale applications, there are also small-scale applications that use RICs, such as the radioisotopic thermoelectric generators (RTGs)^17^ which are often installed in space-bound objects that require energy. They have been supported by NASA^18^ for spaceflight missions^19^ and recently for the power generation on Mars^20^ because they are considered particularly reliable, mainly due to their light weight and compact design^21^. All these factors make RICs the ideal heating source for the POP system, especially that solar power on Mars is inefficient and the POP system must operate for long periods of time without human assistance.

For the perovskite material, Vieten et al.^22,23^ experimentally and theoretically investigate a large number of suitable perovskites for two-step solar thermochemical redox cycling, which are usefully employed to study the equilibrium curves to determine the appropriate temperate properties profile for this application. This work therefore investigates the feasibility of this novel idea. This includes identifying perovskite material suitable for the thermochemical process which will not form stable carbonates, defining the boundary conditions for a system as an alternative to an upscaled MOXIE, conceptualizing the design, and optimizing for important variables such as total weight and primary thermal performance. Several interesting aspects that require additional research are also mentioned, and recommendations for further future work are provided.

## System methodology

The POP system relies on the underlying chemical process that is based on the temperature-dependent chemical potential of oxygen on multivalent metal oxides. Such metal oxides are for example perovskites (*AB*O_3_), where *A* and *B* are two different metals. In the oxidized state *AB*O_3_, it releases oxygen in thermodynamic equilibrium when the temperature is increased and/or the oxygen partial pressure is lowered with reduction to the reduced state *AB*O_3-*δ*_, where *δ* is the oxygen non-stoichiometry:

1 $$\begin{array}{*{20}c} {AB{\text{O}}_{3} \to AB{\text{O}}_{3 - \delta } + \frac{\delta }{2}{\text{O}}_{2} .} \\ \end{array}$$

The reduced metal oxide can then absorb oxygen again at lower temperature and/or higher oxygen partial pressure and is thus re-oxidized:

2 $$\begin{array}{*{20}c} {AB{\text{O}}_{3 - \delta } + \frac{\delta }{2}{\text{O}}_{2} \to AB{\text{O}}_{3} .} \\ \end{array}$$

The reduction and oxidation lead to a formation or filling of oxygen vacancies, respectively.

The temperature of the oxidation step is typically between 250 and 700 °C, the reduction step is performed at 500–1000 °C. The driving force of this redox process is the Gibbs free energy (Δ*G*) which depends on the material specific enthalpy (Δ*H*) and entropy (Δ*S*) and can be influenced by the process parameters oxygen partial pressure ($p_{{{\text{O}}_{2} }}$) and temperature (*T*):

3 $$\begin{array}{*{20}c} {\Delta G = \Delta H^\circ - T\Delta S^\circ + \frac{1}{2}RT\ln \left( {\frac{{p_{{{\text{O}}_{2} }} }}{p^\circ }} \right),} \\ \end{array}$$

where R is the gas constant and *p°* is the reference oxygen partial pressure. The symbol ° shows that the value is at standard pressure.

The temperatures and oxygen partial pressures under which the reactions (1) and (2) take place are determined by the thermodynamic properties of the respective metal oxide, in particular by its redox enthalpy^11,22^. The practical lower limit of the oxidation temperature is determined by the oxidation kinetics^11,12,24^ of the corresponding material, since at too low temperatures the reaction would proceed too slowly for technical feasible application. Moreover, the lower temperature limit is defined by practical considerations, such as the time it takes to cool the material to this temperature level vs. an increased amount of redox cycles per time unit.

This characteristic is exploited by periodically cycling oxidation and reduction in the following way:

- After oxidation, of the perovskite in the Martian atmosphere at lower temperatures, the perovskite heats up and releases some of its oxygen, according to the equilibrium ${\Delta }G$ (3). It has thus reached its final reduction state.
- After reduction, the perovskite is exposed to the Martian atmosphere and consequently cools down. At lower temperatures and the oxygen partial pressure on Mars, it absorbs oxygen into its structure at equilibrium, until it has reached its final oxidation state.

Note that the oxygen yield is determined by the difference ∆*δ* in the non-stoichiometry of the perovskites.

**Figure 1:**
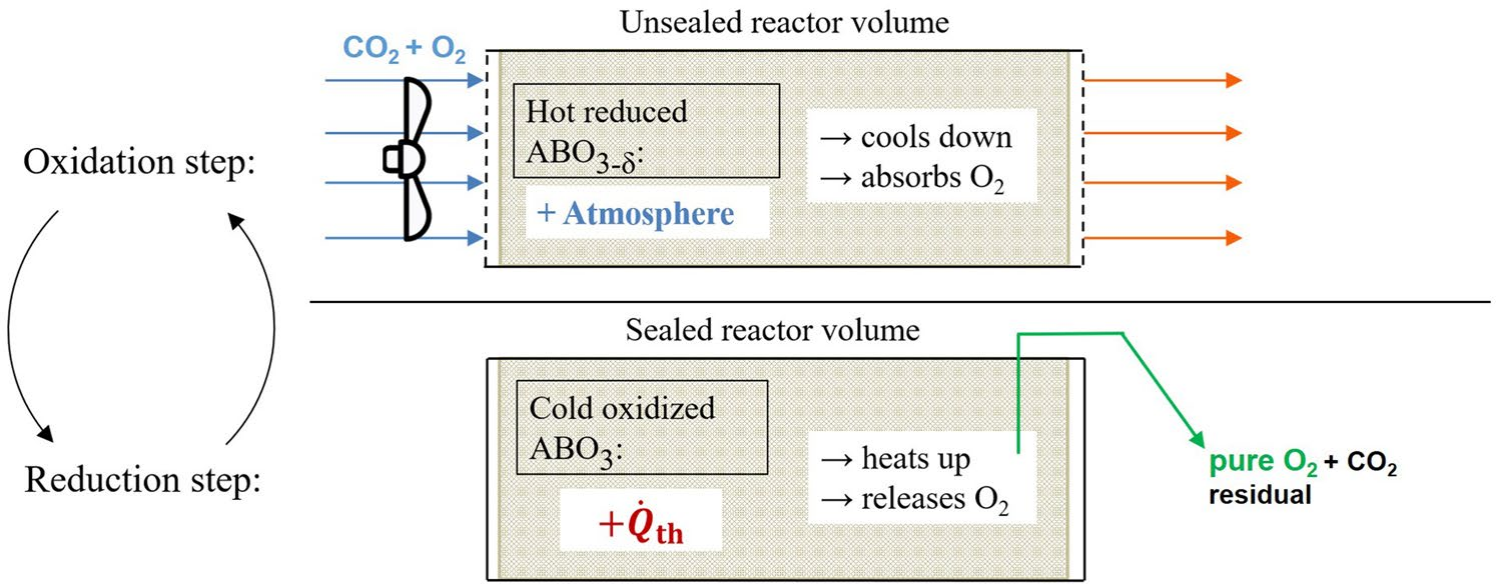
Visualization of the operating principle of the POP system. The oxidation step is carried out in an unsealed reactor, which leads to cooling of the perovskite with simultaneous oxidation of it. The reactor is then sealed, the temperature rises due to radioactive decay and oxygen is released as a result.

The main task of the POP system described in this paper is to execute these redox processes for thousands of cycles, as shown in the visualization of the operating principle of the POP system in Fig. 1.

**Figure 2:**
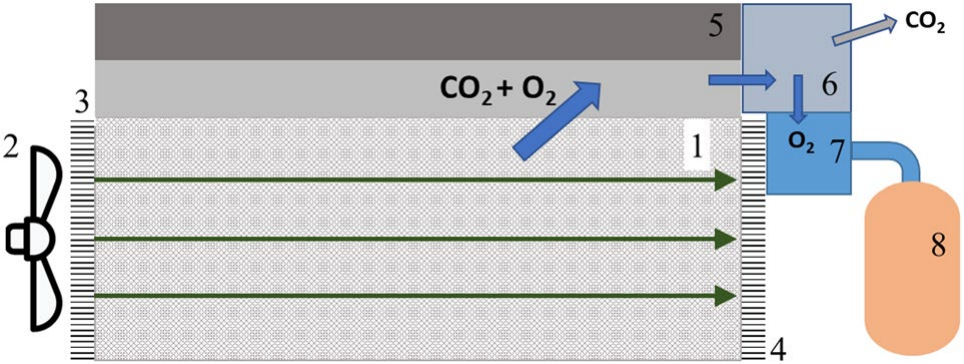
POP-system components. Adapted from^25^.

However, to realize this technically, the full system (shown in Fig. 2) must include the following components^25^:

1. *Composite material*: The *AB*O_3_-RIC (radioisotope compound) is arranged in a structure in such a way, that a good gas flow through the material is possible and kinetic gas exchange is maximized.
2. *Blower*: The blower at the inlet of the device provides sufficient flow of Martian atmosphere through the redox material so that the residual oxygen from the atmosphere oxidizes the redox material.
3. *Inlet seal and (4) outlet seal*: They enable the reaction chamber filled with composite material to be sealed off from the atmosphere in a largely gas-tight manner. This allows the perovskites to reheat and release oxygen.
4. *Thermoelectric device*: Electrical energy for the operation of pumps and compressors is generated from the temperature difference between the exhaust stream and the Martian atmosphere. If necessary, it might also be utilizing some heat directly from the reactor chamber.
5. *CO*_*2*_* separation device*: Separates the unwanted leftover CO_2_ from the gaseous mixture after the reactor chamber is sealed.
6. *Liquefaction device*: Liquefies the oxygen (and some insignificant parts of other gases like nitrogen and noble gases) for higher density.
7. *Storage tank*: Zero-boil off light weight tanks store the oxygen.

Further description of (6): A lot of possible process options to separate the CO_2_ from the gaseous phase could be thought of, e.g. membrane separation, compression until CO_2_-liquefacation or compression and subsequent expansion. As the second option does not benefit the following device (7) and it, as well as the third option, would be power intensive without further advantages, the use of a membrane seems quite appropriate. Elsewise a pump could suck out the leftover atmosphere from the reaction chamber (without any compression) in the first few seconds, as the heating up takes some time and the oxygen release does not run fully right away.

## Boundary conditions

In the following, the boundary conditions of the Martian atmosphere, for the volumetric flow rate and for the material selection are presented.

### Martian atmosphere

Based on the analysis of measurements from the Mars rover ‘Curiosity’, it was found that the Martian atmosphere contains a volumetric oxygen content ($\varphi_{{{\text{O}}_{2} }}$) of 0.16% at a mean total surface pressure ($p_{{\text{M}}}$) in Gale Crater of 846 Pa, resulting in an oxygen partial pressure ($p_{{{\text{O}}_{2} }} )$ of 1.36 Pa^16,26^. For reasons not yet explained, the oxygen content shows substantial interannual variability, with a seasonal variation of 13% from the mean value^26^. Further information on the annual mean volume mixing ratio of the Martian atmosphere measured by ‘Curiosity’ is summarized Table 1, which is corrected and fitted for annual mean conditions.

**Table 1:** Annual mean volume mixing ratios for the Martian atmosphere^26^.

Atmospheric component	CO_2_	N_2_	Ar	O_2_	CO
Annual mean mixing ratio	0.951	0.0259	0.0194	0.00161	0.00058
Seasonal variation from mean	1%	10%	9.7%	13%	36%

It is important to note that the rover ‘Curiosity’ landing site in Gale Crater is more than 4000 m below topographic datum^27^. This has a significant implication for the atmospheric surface pressure and density, ranging from a low of 30 Pa on Olympus Mons to over 1155 Pa on Hellas Planitia (7152 m below the topographic datum)^28^. The altitude of the landing site should therefore be taken into account, and Golombek et al.^29,30^ indicate that a Mars mission could indeed target areas where the altitude is about 4000 m below the topographic datum. Since the surface pressure changes significantly over the course of a Martian year, it must be taken into consideration in all final designs^16^.

In addition, the thin Martian atmosphere can only store a small amount of solar heat, resulting in relatively large daily atmospheric temperature differences above the surface and seasonal fluctuations in daily mean temperatures. For this reason, and because location has a significant effect on the angle of incidence of the sun and thus on solar irradiance and surface temperature, the mean conditions of the Martian atmosphere, summarized in Table 2, are used to simplify the calculations, especially since the landing site is undefined until now.

**Table 2:** Mean Martian atmosphere conditions used in calculations^31^.

Quantity	Symbol	Value
Temperature	$T_{{\text{M}}}$	210 K
Total pressure	$p_{{\text{M}}}$	846 Pa
Oxygen ratio	$\varphi_{{{\text{O}}_{2} }}$	0.16%
Oxygen partial pressure	$p_{{{\text{O}}_{2} }}$	1.36 Pa

For later work on the POP system, more accurate values for atmospheric temperatures are needed. It is recommended to obtain local (both temporal and spatial) climate data after a landing site has been selected, e.g., using the Mars Climate Database model^32,33^. This open-source tool allows modeling of a wide range of atmospheric properties.

### Volumetric flow rate for the production of oxygen

The mean conditions of the Martian atmosphere combined with the goal of producing 2.25 kg of oxygen per hour ($\dot{m}_{{{\text{O}}_{2} }}$) form the fixed boundary conditions for the required oxygen-containing volumetric flow. In this study, two ratios are assumed. One represents an ideal case (i) 80% of the oxygen is absorbed from the inlet flow ($n_{{{\text{O}}_{2} ,{\text{abs}}}} = 0.8$) and one represents a worst case (ii) 20% of the oxygen is absorbed from the inlet flow ($n_{{{\text{O}}_{2} ,{\text{abs}}}} = 0.2$). This paper concentrates on the ideal case, i.e. it assumes that the kinetics of oxidation are fast enough to reach an oxygen uptake rate of 80%. The detailed results using the low oxygen uptake, i.e. 20%, are given in the supplementary information. Due to the lack of information on the kinetics of the various materials, these values are an uncertain estimate.

Under these assumptions and using the ideal gas law, the mean volumetric flow rate is given by

4 $$\dot{V} = \dot{V}_{{{\text{O}}_{2} }} = \frac{{{\text{R}} \cdot \frac{{\dot{m}_{{{\text{O}}_{2} }} }}{{M_{{{\text{O}}_{2} }} }} \cdot T_{{\text{M}}} }}{{p_{{{\text{O}}_{2} }} }} \cdot \frac{1}{{n_{{{\text{O}}_{2} ,{\text{abs}}}} }} = 31.3 \,{\text{m}}^{3} /{\text{s}}$$

where $M_{{{\text{O}}_{2} }} :$ molar mass of oxygen, $T_{{\text{M}}} :$ mean Martian temperature, $n_{{{\text{O}}_{2} ,{\text{abs}}}} :$ fraction of oxygen absorption is a subsequent boundary condition and can be changed after the landing site is defined and the concerning temperature is known.

Since the atmospheric volume containing the required oxygen can only be blown in during the oxidation time step $\left( {t_{{{\text{ox}}}} } \right)$, the volumetric flow rate during oxidation ($\dot{V}_{{{\text{ox}}}} )$ can be defined as

5 $$\begin{array}{*{20}c} {\dot{V}_{{{\text{ox}}}} = \frac{{\dot{V}_{{{\text{O}}_{2} }} }}{{n_{{{\text{cycle}}}} \cdot t_{{{\text{ox}}}} }} = \dot{V}_{{{\text{O}}_{2} }} \cdot \frac{{t_{{{\text{red}}}} + t_{{{\text{ox}}}} }}{{t_{{{\text{ox}}}} }}} \\ \end{array}$$

where $n_{{{\text{cycle}}}}$ refers to the number of cycles per hour.

### Material selection

**Figure 3:**
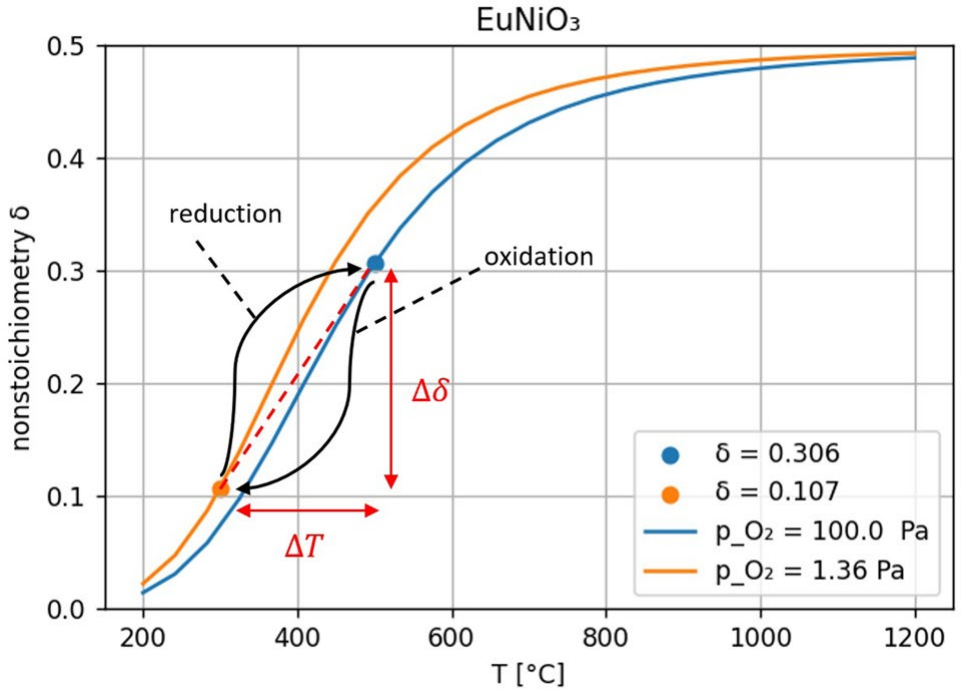
Two non-stoichiometry equilibrium curves of EuNiO_3_perovskite, end points of reduction and oxidation reactions, temperature and non-stoichiometry delta values.

In each redox cycle, the oxygen yield is determined by the difference ∆*δ* of their non-stoichiometry. The compound-specific equilibrium curves of each perovskite are the main criteria for suitability in a POP system. Thereby, a high difference quotient $\Delta \delta$ (the “yield”) over the temperature change ($\Delta T$) (the “price”) is preferred. Here, the temperature dependent oxidation kinetics define the oxidation temperature. Figure 3 shows equilibrium curves based on theoretical data^22,23^ for the perovskite EuNiO_3_ as an example.

As for a first assumption the reduction occurs with a closed chamber, the equilibrium oxygen partial pressure is set to 100 Pa (0.001 bar). This reduction pressure is chosen according to Brendelberger et al.^34^ which exploits the optimum efficiency of both mechanical and thermochemical pumps. For the oxidation, the mean Martian oxygen partial pressure is used. The points (shown in Fig. 3) highlight possible final operating conditions for each step resulting in a temperature swing accompanied by a change in non-stoichiometry.

Although, the thermal stability of the perovskites (*AB*O_3_) is determined by the cations at position A and B , another important requirement for the material used is the prevention of the formation of carbonates, which hinder the cyclic and reversible redox reaction of the metal oxide. Since the Martian atmosphere consists largely of carbon dioxide, metals that form stable carbonates at the corresponding operating temperatures are out of question. These are essentially perovskites with alkali/alkaline earth metals at one of the lattice positions: position A or position B^35^. Lanthanides, actinides and transition metals, on the other hand, do form carbonates, but most of them decompose at relatively low temperatures, so they do not interfere with the redox reaction^36^.

To sum it up, the redox material for the concentration of oxygen needs to fulfil the following requirements:

1. Fast oxidation kinetics.
2. $$\Delta \delta { }$$ ≥ 0.1 for a given $$\Delta T$$.
3. No formation of stable carbonates and oxalates.

## Design approach

A suitable reaction chamber for the POP system is required to achieve the primary goal of producing 2.25 kg of oxygen per hour under the extreme environmental conditions on Mars, as the POP system is intended to operate in all seasons and at all times on Mars; therefore, an initial best-case design is presented in this section. To achieve this, a reactor cross-sectional area $\left( A \right)$ of approximately $1.13\,{\text{ m}}^{2}$ is required for an oxidation–reduction step time of 15 min and a maximum flow velocity $(v_{0} )$ of $55$ m/s at the reactor inlet. These values were calculated using the parameters and correlations listed in Table 3.

**Table 3:** Parameters and correlations used to calculate reactor cross-sectional area.

Parameter	Symbol	Value	Equation	
Oxidation step time	$t_{{{\text{ox}}}}$	15 min		
Reduction step time	$t_{{{\text{red}}}}$	15 min		
Cross-sectional area	$A$	1.13 ${\text{ m}}^{2}$	$\frac{{\dot{V}_{{{\text{ox}}}} }}{{v_{0} }}$	(6)
Adiabatic index of CO_2_	$\kappa_{{{\text{CO}}_{2} }}$	1.23	$\frac{{c_{{\text{p}}} }}{{c_{{\text{v}}} }}$	(7)
Speed of sound in martian atmosphere (100% CO_2_)	$c_{{{\text{CO}}_{2} }}$	221 m/s	$\sqrt {\kappa_{{{\text{CO}}_{2} }} \cdot \frac{{R \cdot T_{{\text{M}}} }}{{M_{{{\text{CO}}_{2} }} }}}$	(8)
Maximum flow velocity	$v_{0}$	55 m/s	$M \cdot c_{{CO_{2} }}$	(9)

To avoid significant flow compression, turbulence, and consequently higher blower power, the flow velocity was limited to a Mach number (*M*) of 0.25. A typical value for assuming incompressible flow is 0.3, but the boundary layers of the geometry will accelerate some parts of the flow.

Assuming that the Martian atmosphere is 100% CO_2_, the sound velocity equation derived from the ideal gas law (8) was used, with the mean temperature of Mars ($T_{{\text{M}}}$) applied. Although the stream has higher temperatures when leaving the reactor chamber, this only increases the speed of sound and thus the Mach number, which is why $T_{{\text{M}}} = 210 {\text{K}}$ is used in Eq. (8).

Since the required cross-sectional area $\left( A \right)$ calculated is relatively large, it must be reconciled with several other factors, namely:

- The pressure drop between the inlet and the outlet of the apparatus should be as low as possible, as it determines the blower performance.
- The kinetic contact between the gaseous stream and the perovskite surface must be sufficient for the absorption kinetics. Therefore, laminar flow with thick boundary layers must be avoided since impulse exchange and lateral flow velocities are minimal.
- The perovskite must be cooled down to the desired target temperature $T_{ox}$, which determines $t_{{{\text{ox}}}}$. Throughout the reaction chamber, the temperature should be nearly homogeneous, as hot spots lead to lower stoichiometry and cold spots lead to slower kinetics.
- The reactor and its internal structure must maintain structural integrity: That is, it must:
  1. Be mechanically stable at the highest loads during rocket launch (several *g*).
  2. Withstand thermal expansion stresses for thousands of cycles.
  3. Be abrasion resistant to some degree, since the blown volume will most likely contain some particles of Martian dust that could become impacting projectiles at the high flow velocities.
- All the materials used to build the structure must be heated by decay heat of the radioisotopes and should therefore be minimized.

**Figure 4:**
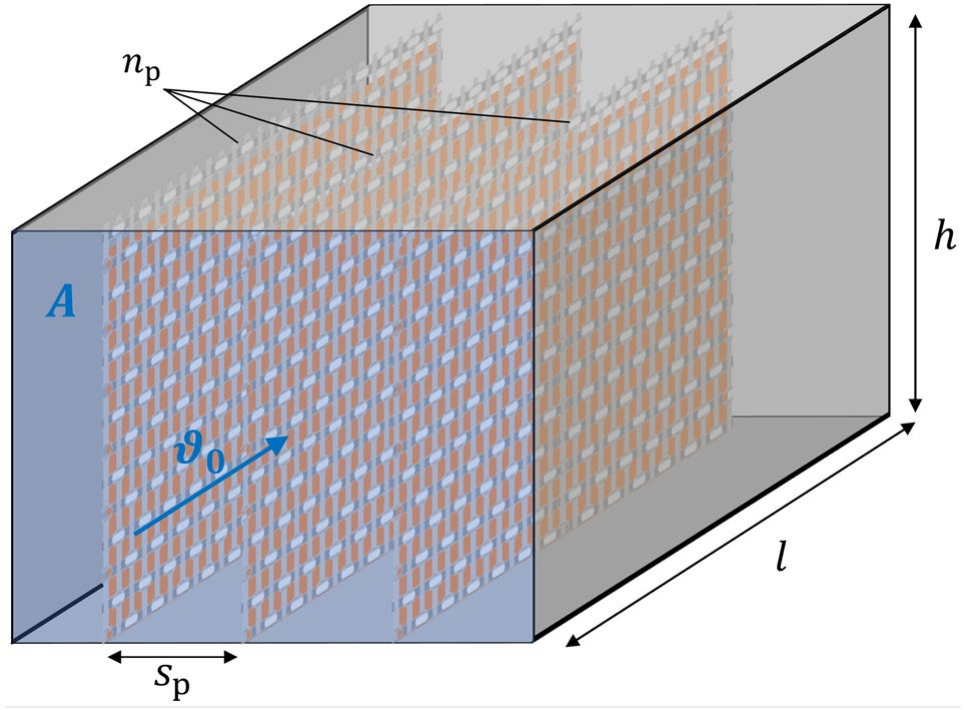
Reactor chamber structure with thin curtains: two carbon fiber fabrics enclosing a perovskite-radioisotope compound granule; multiple curtains flowed longitudinal, and; chamber walls made of metal.

Figure 4 shows a geometry of parallel arranged plates with $n_{{\text{p}}}$ plates, gap width $(s_{{\text{p}}} )$, height $(h$) and length $\left( l \right)$ enclosed inside a reactor chamber, with an inlet- and an outlet-seal, that should enable the reaction chamber filled with composite material to be sealed off from the atmosphere in a largely gas-tight manner.

The cross-sectional area $\left( A \right)$ at the inlet in relation to the design variables is given by

10 $$\begin{array}{*{20}c} {A = n_{{\text{p}}} \cdot s_{{\text{p}}} \cdot h} \\ \end{array}$$

These design variables have conflicting dependencies on aspects important to the POP system optimization process, such as pressure drop, temperature homogeneity, mechanical stability, etc., as shown in Table 4.

**Table 4:**
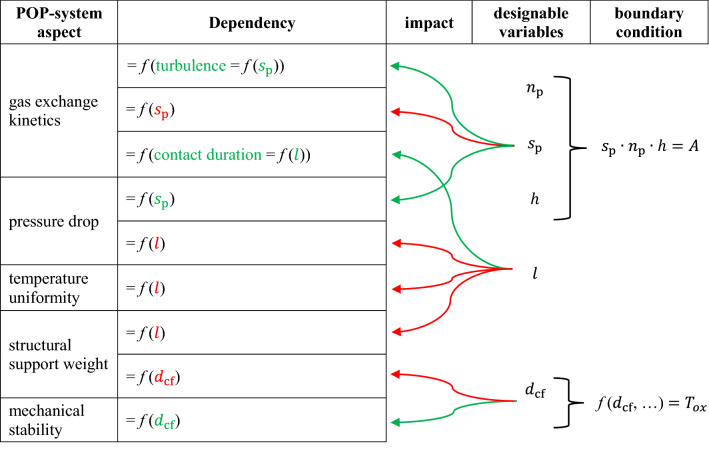
Some interdependencies between reactor chamber variables and important aspects of the POP system.

*f* indicates that a “dependency” exists, even if it cannot be quantified. If a variable is green, it means that increasing its value is beneficial for the corresponding aspect of the POP system, red means the opposite.

So, for example, if one increases the length $l$ of the plates:

- Increases the residence time of an oxygen particle in the plate geometry (which benefits the absorption possibility and thus the kinetic exchange),
- Increases the pressure drop and thus the power consumption of the blower (detrimental),
- Increases the non-uniformity of the temperature profile at the end of the oxidation step (a cooler tip and a hotter end of the plates are disadvantageous),
- Increases the overall weight.

Table 4 does not give a complete overview of all the interdependencies of the design options, but shows that there is no simple optimization for the competing aspects and therefore experimental work and experience are necessary.

The afore mentioned requirements for resistance against abrasion and the ability to withstand thermal expansion stresses cannot be reduced to a variable and are therefore discussed separately.

The RIC (e.g. PuO_2_) gets mixed with the perovskite (e.g. EuNiO_3_). Since the coefficients of thermal expansion are likely to be very different, any ceramic structure will eventually crumble or break. Even if equalization of the thermal expansion coefficients could be ensured, rigid ceramic plates with a thickness of $1 \mathrm{mm}$ or less would be fragile and fraught with risks.

**Figure 5:**
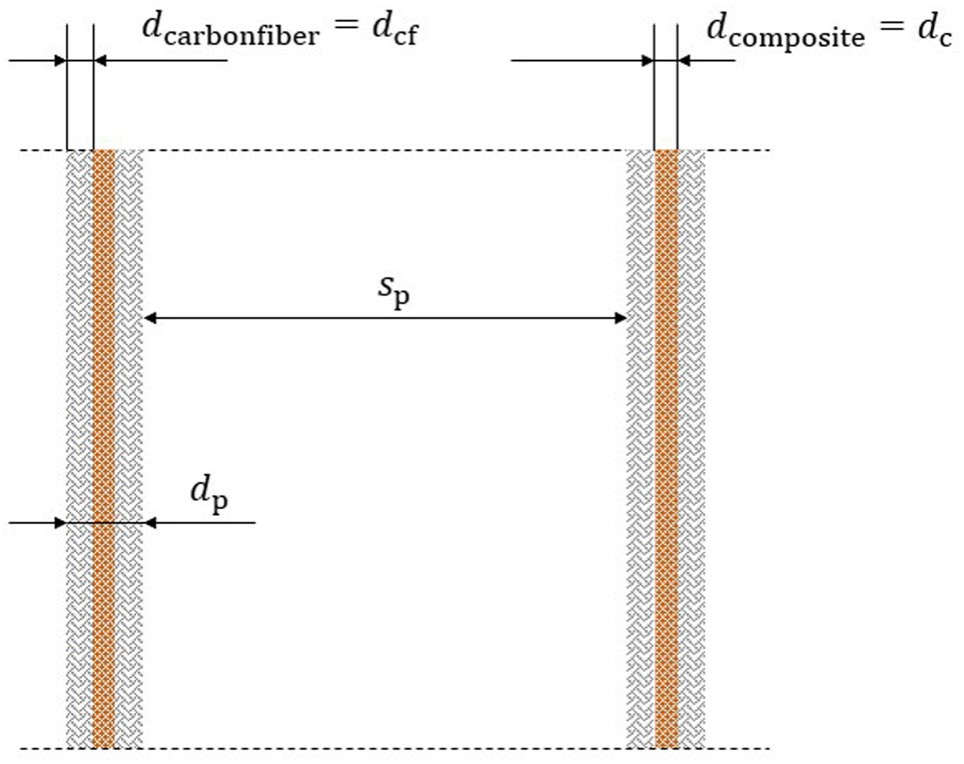
Curtain cross section.

Therefore, load-bearing structural material is needed that accommodates the RIC and *AB*O_3_, allows gas exchange, and is as lightweight as possible. Therefore, the structural design shown in Figs. 4 and 5 is considered suitable for this purpose.

In this design, the *AB*O_3_ and RIC crumbs or powder are sandwiched between two carbon fiber structures.

The ceramic materials can be supplied as granules and the perovskite should be in a form that maximizes the active surface area to ensure rapid kinetic gas exchange. A type of foam ceramic with a structure like activated carbon would be ideal.

Carbon fibers are selected for their high mass-specific strength and high working temperature. When coated with Al_2_O_3_, the fibers can withstand oxidation temperatures up to 800 °C^37^. Alternatively, ceramic fibers such as WHIPOX™, wound highly porous oxide composite, are a high temperature stable fiber reinforced oxide ceramic, a brittle electrically insulating material that can be operated at temperatures up to 1400 °C and has excellent resistance to thermal cycling^38,39^. Ideally, a fabric pattern would retain the crumbs of both compounds inside while guaranteeing maximum gas permeability. If necessary, such thin fabric “curtains” could be supported and fixed, for example, by lateral rods.

With such a fabric-like fiber structure, it is important to optimize the grain size of the two compounds it contains, because the smaller the crumbs, the better the heat conduction, and the larger the crumbs, the coarser the fabric can become. Such a fabric pattern should ensure adequate resistance against dust particle bombardment. Nevertheless, it must retain its gas permeability and not become clogged with dust particles even after many cycles.

To simplify the calculations, the enclosures are represented by two solid plates and it is assumed that a thickness of $d_{{{\text{cf}}}} = 1 {\text{mm}}$ is sufficient.

A gap width of $s_{{\text{p}}} = 3 {\text{cm}}$ is chosen as this leads to a relatively low pressure drop per length and at least some kind of turbulent behavior. A Reynolds number of

11 $$\begin{array}{*{20}c} {Re_{{{\text{channel}}}} = \frac{{2 \cdot \rho \cdot s_{{\text{p}}} \cdot v_{0} }}{\eta } = 6267.} \\ \end{array}$$

corresponds to a flow that is no longer purely laminar ($Re_{{{\text{channel}},{\text{lam}}}} \approx 2300$), and not yet fully turbulent ($Re_{{{\text{channel}},{\text{turb}}}} \approx 10,000$) and therefore in transition^40^. For the density $\left( \rho \right)$ and the dynamic viscosity ($\eta ),$ the values of CO_2_ under Martian conditions are used^41^.

It should be noted that the gap width in particular is a highly arbitrary choice and should not be considered profound or definitive. The gas exchange in terms of width must be validated experimentally. In particular, this gap width has a direct effect on the assumed oxygen absorption rate of 80%, since a large portion of the gas flow passes without direct contact with the perovskite material, resulting in a significant reduction in the oxygen absorption rate by the perovskite material. The same applies to the length and height of the chamber geometry. The intentional turbulence inducing by flow disrupting elements could also be an option if experiments show that only oxygen is absorbed from the boundary layers. Higher turbulence leads to higher lateral momentum and gas exchange at the price of a higher pressure drop and could increase absorption.

### Electric power: electric blower and separation units

The electrical power required to operate the POP system is a combination of electricity needs of several components: electric blower, CO_2_ separation (via pre-liquefaction compression unit) and O_2_ liquefaction units.

To provide the necessary oxygen volume flow during oxidation (defined in Eq. 5), an electric air blower is needed.

Using Eq. (5), the electric power of the blower ${(P}_{blower})$ and the mean constant electric power of the blower (${\overline{P} }_{\mathrm{blower}})$ can be calculated using Eq. (12) and (13), accordingly.

12 $$P_{{{\text{blower}}}} = \dot{V}_{ox} \cdot \Delta p$$

13 $$\begin{array}{*{20}c} {\overline{P}_{{{\text{blower}}}} = \dot{V} \cdot \Delta p = 1.57 {\text{kW}}} \\ \end{array}$$

These values are determined by the mean pressure drop (∆*p*) from inlet to outlet. In order to determine a suitable ∆*p* value, multiple pressure drops are simulated for different design variants (different channel width and length of the reactor chamber shown in Fig. 4) by performing rough 2D CFD simulations with ANSYS Fluent. The results ranged from 25 to 100 Pa, suggesting that 50 Pa is a reasonable assumption for ∆*p*. However, this estimate should be considered preliminary.

In addition, the oxygen produced needs to be separated from residual Martian atmosphere and liquefied at the outlet to be stored. Johnson et al.^42^ published a detailed estimation of the necessary powers and masses for an oxygen liquefaction system for use on the Martian surface. Assuming the needs to liquefy 2.25 kg/h of pure oxygen at 0 °C at 1 bar, the authors compare different systems in terms of power consumption, mass, cost and other aspects. They assume the most unfavorable conditions in the Martian atmosphere, i.e. the highest surface temperatures (which prevent cooling), and conclude that by using the best option of a Tube-on-Tank architecture (also known as ‘Broad Area Cooling’ or ‘Distributed Refrigeration’), a device with a mass of 68 kg and an electric power consumption of 2.87 kW would be possible.

While this power demand is significant, it should not be overrated with respect to the suitability for the POP system, as any other ISRU system for the production of oxygen on Mars (including an upscaled MOXIE) faces the same necessity for liquefaction.

For the POP system, it is important to lower the oxygen partial pressure during the reduction reaction, as this supports and accelerates the oxygen release and thus significantly improves $\Delta \delta$ [see Eq. (3)] between the non-stoichiometry equilibrium curves of the perovskite. Thereby, the final conditions of the reduction step determine the $\Delta \delta$ responsible for the oxygen yield.

At the same time, lowering the reduction release pressure implies higher electrical consumption to compress the released gas to 1 bar for subsequent liquefaction. There is thus a balance between power consumption of the pump and the oxygen release of the perovskite, with the optimal pressure changing together with the changing $\delta$ in $AB{\text{O}}_{3 - \delta }$ as well as with the temperature over the course of the reduction reaction.

For a first simplified estimation, the following assumptions are made in this work: The pressure at the beginning of the reduction step is 0.01 bar and decreases linearly to 0.001 bar at the end of the step; the power consumption is calculated as having to pump a constant pressure of 0.005 bar. The oxygen is cooled to 0 °C before and after compression by heat exchange with the ambient air.

The required electrical power is estimated for isentropic compression of an ideal gas in the Supplementary Information. A compressor efficiency of 60% results in a power of 841 W. If the compression is divided into two steps (0.005 to 0.07 to 1 bar) and cooled to 0 °C in between, the power can be reduced to 566 W.

The compression power is necessary during the reduction step, but is calculated as an hourly average like the liquefaction power estimate from^43^. Thus, the mean power consumption required to store the released oxygen in liquid form is estimated to be 3.57 kW, which is the sum of the average electrical power for isentropic compression (0.7 kW) and electric power of the Tube-on-Tank option (2.87 kW). Table 5 gives a summary of the power requirements for the operation of the various components of the POP system.

**Table 5:** Power consumption of the POP system various units to produce 2.25 kg/h O_2_.

Power consumptions	Value (kW)
Electric Blower	1.57
Compressor (60% efficiency)	0.841
Two steps Compressor	0.566
Mean Isentropic Compression	0.7
Tube-on-tank	2.87
Mean value for liquid oxygen storage	3.57

### Utilization of waste heat—heat from exhaust stream

Each oxidation can be utilized to generate power by using the waste heat. The total thermal power calculated as a mean over the oxidation time is:

14 $$\begin{array}{*{20}c} {\dot{Q}_{{{\text{ox}}}} = \dot{Q}_{{{\text{th}}}} + \frac{{\Delta Q_{{\text{m}}} + \Delta Q_{{\text{r}}} }}{{t_{{{\text{ox}}}} }}.} \\ \end{array}$$

Thereby, $\Delta Q_{{\text{m}}}$ is the change of the heat of the material between the reduction and oxidation temperature, $\Delta Q_{{\text{r}}}$ is the heat due to the exothermic oxidation and $\dot{Q}_{{{\text{th}}}}$ is the heat produced by the ongoing decay of the radioisotopes. Especially important for the exergetic quality of $\dot{Q}_{{{\text{ox}}}}$ is the temperature at which the gas is released to the Martian atmosphere, i.e. the end temperature of the stream, or rather the corresponding temperature difference. Assuming 100% CO_2_, ideal gas law and therefore pressure and density independent enthalpy change as well as the temperature independence of the heat capacity $c_{{{\text{p}},{\text{CO}}_{2} }}$, we can estimate that mean difference through

15 $$\begin{array}{*{20}c} {\Delta T_{{{\text{CO}}_{2} }} = \frac{{\dot{Q}_{{{\text{ox}}}} }}{{\dot{m}_{{{\text{CO}}_{2} }} \cdot c_{{{\text{p}},{\text{CO}}_{2} }} }}} \\ \end{array}$$

where

$$\dot{m}_{{{\text{CO}}_{2} }} = \frac{{p_{{\text{M}}} \cdot \dot{V}_{{{\text{ox}}}} }}{{R \cdot T_{{\text{M}}} }} \cdot M_{{{\text{CO}}_{2} }}$$

An efficiency

16 $$\begin{array}{*{20}c} {\eta_{{\text{util }}} = \frac{{P_{{{\text{blower}}}} }}{{\dot{Q}_{{{\text{ox}}}} }} } \\ \end{array}$$

that compares the exhaust stream’s waste heat flux with the electric consumption of the blower gets introduced, as these two energy fluxes occur simultaneously.

Due to the power consumption of the liquefaction process an additional electric power device is required.

## Results and discussion

The POP system collects oxygen by performing two redox processes over thousands of cycles by forcing convection across the surface of the partially reduced internal perovskite structure to absorb oxygen into the material, reducing it by heating the material with RIC, pumping out the mixture of released oxygen and some residual atmosphere (CO_2_), separating it, liquefying the high-purity oxygen and storing it in suitable zero-boil-off tanks. Therefore, identifying suitable materials—radioisotopes (RI), the corresponding RIC, perovskites and possibly other structural materials—is key to the performance of the POP system. The free variables i.e. oxidation and reduction temperature and time are optimized and the best-case is presented here. Critical aspects of the technology as well as the weaknesses and uncertainties are highlighted, and further investigation paths are provided.

### Materials

For the presented process, the redox material as well as the heating material needs to be selected. In the following, the criteria and the selection for both materials are discussed.

### Redox material

In the Material Selection three major selection criteria are presented. To satisfy the first requirement, the minimum oxidation temperature is set to 300 °C, as Bulfin et al.^12^ indicates that the kinetics slow down at lower temperatures, meaning the oxidation proceeds too slow for technical applications. Although Bulfin et al.^12^ investigated SrFeO_3_, we take the work’s results since no experimental data on kinetics or oxygen diffusion are available for many perovskites yet.

To evaluate the second criteria, the theoretical equilibrium data from Vieten et al.^44^ are analyzed (see section on Material Selection) according to their enthalpy. The enthalpy influences the oxygen affinity and therefore, also the required reduction temperature. An ideal perovskite is reduced at relatively low temperature, but is still easily re-oxidized.

Promising perovskites such as EuCuO_3_, EuNiO_3_, LaAgO_3_, Sm_0.5_La_0.5_CoO_3_, and Sm_0.5_La_0.5_NiO_3_ are used for the investigation. The results of the equilibrium curves for the different perovskites can be found in Fig. 1 of the Supplementary Information and in Fig. 3. These perovskites are examined to determine whether they form stable carbonates and oxalates under the experimental conditions. We define stable as compositions which are formed at the oxidation temperatures or lower and do not decompose until the reduction temperature is reached. Both europium oxalate (Eu_2_(C_2_O_4_)_3_)^45^ and nickel carbonate (NiCO_3_)^46^, which may form as a by-product during the redox cycling of EuNiO_3_, are unstable according to this definition. Selecting a temperature swing between 300 and 500 °C, accompanied with a respective oxygen partial pressure swing between 1.36 Pa and 100 Pa (see Fig. 3), the change in non-stoichiometry is $\Delta \delta = 0.199$. Thus, EuNiO_3_ fulfils the second criteria and is selected as the redox material for the following investigation.

### Heating material

Radioisotopes are considered as the heat source, as they have been used frequently in space missions for power generation (RTG). To avoid unreasonable shielding requirements, a short mean free path of the radiation is required. Both α- and β- particles have a short mean free path, contrary to γ and neutron (n) emissions. Moreover, free neutrons can activate stable isotopes to become radioactive. Therefore, radioisotopes with α- or β-decays are preferred. On this basis, possible candidates are identified and discussed.

^238^Pu is used in space missions for decades^40^. It shows 100% α-decay and advantageously low n- and γ- emissions. The neutrons are released due to (α, n) reactions and the spontaneous fission of ^238^Pu. The main source of the γ-radiation is the α-decay. The accompanied reactions only have a minor contribution to the total γ-radiation^40^. Shielding and radiation safety analysis are discussed further in ref.^47^. Since ^238^Pu is not a by-product of nuclear power plants, it has the disadvantage of limited availability. The possible annual production in the USA is estimated to be around 1.5–5 kg^48^. The supplies from the past decades have been mostly spent^48^. Therefore, and due to the limited availability of ^238^Pu and its popular use in space applications, various interested parties compete for this isotope^48^. Similar to its use in radioisotope thermoelectric generators (RTG), ^238^Pu would be embedded in PuO_2_ for the application in the presented POP-system. It has the longest half-life of the presented isotopes (see Table 6), which on the one hand decreases the decay heat, but makes it more suitable for long-term applications.

**Table 6:** Weight-specific thermal powers of the presented radioisotopes and their compounds. A 100% radioisotopic purity is assumed.

	^238^Pu	^90^Sr	^244^Cm
$\dot{q}_{{{\text{RI}}}} = \dot{q}_{{{\text{Isotope}}}} \left[ {\frac{{\text{W}}}{g}} \right]$	$0.57$	$0.92$	$2.78$
$\dot{q}_{{{\text{RIC}}}} = \dot{q}_{{{\text{Composition}}}} \left[ {\frac{{\text{W}}}{{\text{g}}}} \right]$	$0.50$	$0.55$	$2.53$
$$t_{{{\raise0.7ex\hbox{$1$} \!\mathord{\left/ {\vphantom {1 2}}\right.\kern-0pt} \!\lower0.7ex\hbox{$2$}}}} \left[ {\text{y}} \right]$$	$$87.7$$	$$28.9$$	$$18.1$$

^90^Sr is also used in RTGs. The modes of its decay chain (^90^Sr → ^90^Y → ^90^Z) are 100% β- emitting. Due to the slowing down of the β-particles within the redox material, bremsstrahlung is emitted. The shielding of this harmful radiation is described in ref.^49^ and ref.^50^. One advantage of the material is that it is contained in nuclear waste. Therefore, the production of larger quantities is reasonable. However, the proportion of active ^90^Sr is a disadvantage, because fission product strontium contains only 55 at.% ^90^Sr, which reduces its power density^50^. ^90^Sr would be embedded in SrCO_3_, since this carbonate forms during the process anyway in the CO_2_-rich Martian atmosphere.

^244^Cm has the highest weight-specific power density of the three isotopes (see Table 6) and is a 100% α- emitter. On account of its thermal stability, melting point and producibility, it is used as Cm_2_O_3_^51^. The neutron emission of ^244^Cm_2_O_3_ is 45 times greater than ^238^PuO_2_. Therefore, a higher amount of shielding is required, which leads to an increased weight^52^. Cm is a waste product of nuclear plants. The different half-life of the Cm isotopes leads to an improved weight fraction (wt.%) of ^244^Cm. After 5 years, the proportion increases from 80.3 wt.% to 91.75 wt.%^51^.

The mass specific decay heat $$\dot{q}$$ of the three heating materials mentioned is calculated using the mean energy per decay $$(\overline{Q}_{{{\text{decay}}}}$$) and the half-life ($$t_{{{\raise0.7ex\hbox{$1$} \!\mathord{\left/ {\vphantom {1 2}}\right.\kern-0pt} \!\lower0.7ex\hbox{$2$}}}}$$) from^53^:

17 $$\begin{array}{*{20}c} {a_{{{\text{RI}}}} \left[ {\frac{{{\text{Bq}}}}{{{\text{mol}}}}} \right] = \frac{\ln \left( 2 \right)}{{t_{{{\raise0.7ex\hbox{$1$} \!\mathord{\left/ {\vphantom {1 2}}\right.\kern-0pt} \!\lower0.7ex\hbox{$2$}}}} }} \cdot 6.022e23\frac{1}{{{\text{mol}}}}} \\ \end{array}$$

18 $$\begin{array}{*{20}c} {\widetilde{{\dot{q}_{{{\text{RI}}}} }}\left[ {\frac{{\text{W}}}{{{\text{mol}}}}} \right] = \overline{Q}_{{{\text{decay}}}} \cdot a_{{{\text{RI}}}} } \\ \end{array}$$

and

19 $$\begin{array}{*{20}c} {\dot{q}_{{{\text{RI}}}} \left[ \frac{W}{g} \right] = \frac{{\widetilde{{\dot{q}_{{{\text{RI}}}} }}}}{{M_{{{\text{RI}}}} }}} \\ \end{array}$$

For ^90^Sr, the mean energy per decay of ^90^Sr and ^90^Y are added. All the calculations assume that the isotopes are pure. The results for the isotopes as well as the compounds are presented in Table 6.

As all of the presented materials have advantages and disadvantages, all three isotopes were used in the code to find the optimized masses and values for a POP system. Both the case of 100% active ^90^Sr in the carbonate and one with 60% ^90^Sr were calculated. The cases for 100% active ${}_{ }^{238} {\text{Pu}}$ in PuO_2_ and 95% active ^244^Cm in Cm_2_O_3_ have been calculated.

### Masses and power calculations

To ensure the demanded yield of oxygen, the required perovskite mass ($m_{{{\text{ABO}}_{3} }}$) is

20 $$\begin{array}{*{20}c} {m_{{AB{\text{O}}_{3} }} = M_{{AB{\text{O}}_{3} }} \cdot \frac{{\dot{m}_{{{\raise0.7ex\hbox{${{\text{O}}_{2} }$} \!\mathord{\left/ {\vphantom {{{\text{O}}_{2} } h}}\right.\kern-0pt} \!\lower0.7ex\hbox{$h$}}}} }}{{M_{{{\text{O}}_{2} }} }} \cdot \frac{2}{\Delta \delta } \cdot \left( {t_{{{\text{ox}}}} + t_{{{\text{red}}}} } \right) } \\ \end{array}$$

and the required thermal power ($\dot{Q}_{{{\text{th}}}}$) to heat the redox material from the oxidation temperature to the reduction temperature during the reduction time $t_{{{\text{red}}}}$ is calculated from

21 $$\begin{array}{*{20}c} {\dot{Q}_{{{\text{th}}}} = \frac{{\Delta Q_{{\text{m}}} + \Delta Q_{{\text{r}}} }}{{t_{{{\text{red}}}} }}.} \\ \end{array}$$

whereby $\Delta Q_{{\text{m}}}$ is the energy (per cycle) that is necessary to heat up the perovskite, the radioisotope-compound and the structural support material (carbon fibers) and $\Delta Q_{{\text{r}}}$ is the reaction heat (per cycle) for the endothermic reduction of the perovskite. To calculate the reaction heat, the perovskite-specific redox enthalpy per mole oxygen $\Delta H_{{{\text{O}},{\text{ABO}}_{3} }}$ is used. This in turn determines the necessary amount of RIC according to Eq. (23).

22 $$\begin{array}{*{20}c} {\Delta Q_{{\text{m}}} = \left( {\left( {c \cdot m} \right)_{{{\text{RIC}}}} + \left( {c \cdot m} \right)_{{AB{\text{O}}_{3} }} + \left( {c \cdot m} \right)_{{{\text{cf}}}} } \right) \cdot \left( {T_{{{\text{red}}}} - T_{{{\text{ox}}}} } \right), } \\ \end{array}$$

23 $$\begin{array}{*{20}c} {\Delta Q_{{\text{r}}} = \Delta H_{{{\text{O}},AB{\text{O}}_{3} }} \cdot \frac{{m_{{AB{\text{O}}_{3} }} }}{{M_{{AB{\text{O}}_{3} }} }} \cdot \Delta \delta ,} \\ \end{array}$$

24 $$\begin{array}{*{20}c} {m_{{{\text{RIC}}}} = \frac{{\dot{Q}_{{{\text{th}}}} }}{{\dot{q}_{{{\text{RIC}}}} }}} \\ \end{array} .$$

### Energy optimization

The equations and concepts presented result in an energy optimization problem. For the purpose of clarity, the input variables and their characteristics are presented in Table 7 and the resulting variables in Table 8.

**Table 7:** Input variables and their characteristics.

Classification	Variable	Characteristic
Process related	$T_{{{\text{ox}}}}$, $T_{{{\text{red}}}}$, $t_{{{\text{ox}}}}$,$t_{{{\text{red}}}}$	Varied and optimized
Geometry related	$d_{{{\text{cf}}}}$, $l$, $s_{{\text{p}}}$	Chosen
Material related	$M_{{\text{x}}}$, $c_{{\text{x}}}$,$\rho_{{\text{x}}}$, $\Delta H_{{{\text{O}},{ }AB{\text{O}}_{3} }}$,$\dot{q}_{{{\text{RI}}}}$	Given
Martian atmosphere	$T_{{\text{M}}}$, $p_{{\text{M}}}$,$\varphi_{{{\text{O}}_{2} }}$	Given
Approach velocity	$\vartheta_{0}$	Chosen
Pressure drop	$\Delta p$	Obtained from ANSYS Fluent; *f* ($l$, $s_{{\text{p}}}$, $\overline{\vartheta }$)

**Table 8:** Resulting values for the energy optimization.

Classification	Variable
Masses	${\varvec{m}}_{{{\mathbf{RI}}}}$, $m_{{{\text{RIC}}}}$, $m_{{AB{\text{O}}_{3} }}$, $m_{{{\text{cf}}}}$,${\varvec{m}}_{{{\mathbf{total}}}}$
Geometry related	$n_{{\text{p}}}$, $d_{{\text{p}}}$, $d_{{{\text{comp}}}}$,${\varvec{A}}$,$h$
Blower related	$\dot{V}_{{{\text{ox}}}}$, ${\varvec{P}}_{{{\mathbf{blow}}}}$
Energy values	$\dot{Q}_{{{\text{ox}}}}$, $\dot{\user2{Q}}_{{{\mathbf{th}}}}$
Heat utilization related	$\eta_{{{\text{util}}}}$, $\Delta T_{{{\text{CO}}_{2} }}$

Hereby, the highlighted values of $m_{{{\text{total}}}}$, $m_{{{\text{RI}}}}$, $\dot{Q}_{{{\text{th}}}}$, $A$ and $P_{{{\text{blow}}}}$ are optimized.

The following values were set: $d_{{{\text{cf}}}} = 1 {\text{mm}}$, $s_{{\text{p}}} = 3 {\text{cm}}$, $\vartheta_{0} = 0.25 c_{{{\text{CO}}_{2} }}$ and $l = 1 {\text{m}}$. The first three values were explained in the previous sections while the latter is an unsubstantiated choice. Without experimental studies, it is not possible to validate the assumption that a length of 1 m is sufficient for the oxygen absorption process. Thus, this value has to be regarded preliminary and needs further substantiation in future studies.

**Figure 6:**
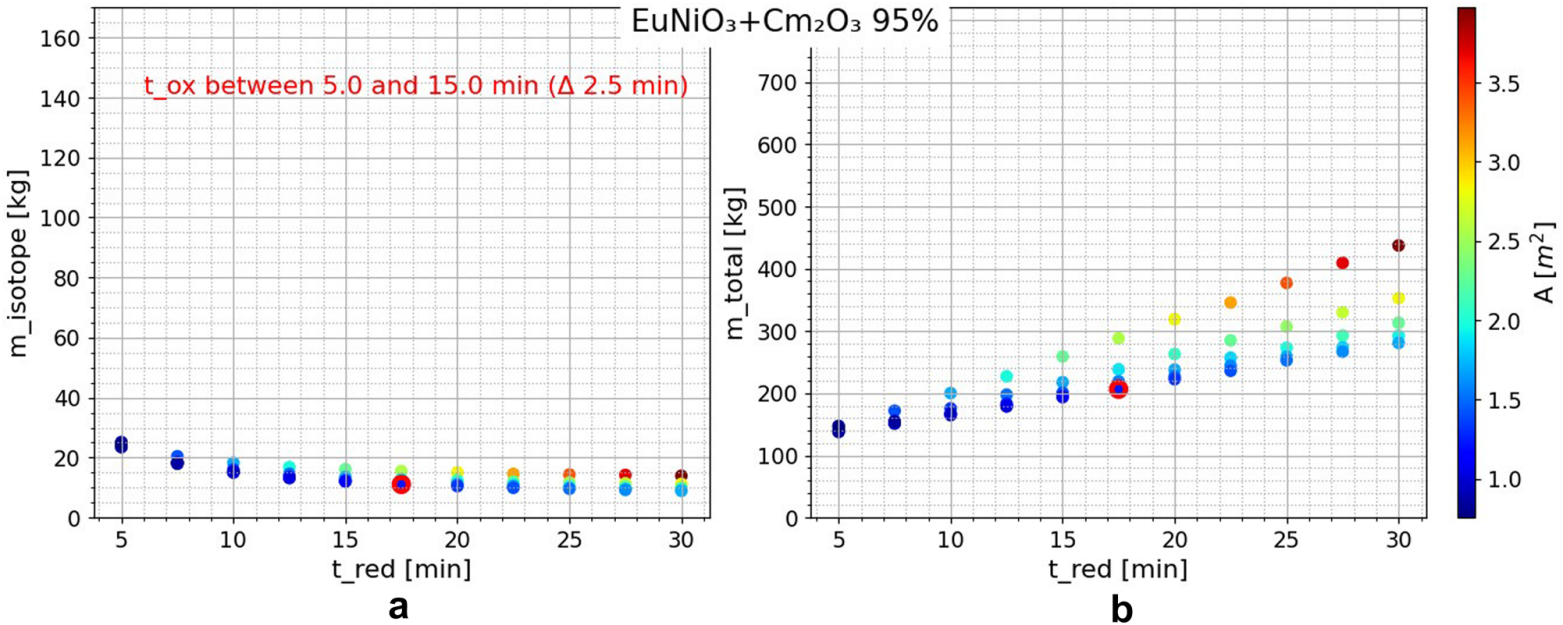
EuNiO_3_-Cm_2_O_3_ (95% ^244^Cm) calculation results for (**a**) isotope mass $m_{{{\text{RI}}}}$, (**b**) total curtain mass $m_{{{\text{total}}}}$, (color) reactor chamber cross-sectional area $A$ over $t_{{{\text{ox}}}}$ (projected axis) and $t_{{{\text{red}}}}$ (x-axis) variation; $T_{ox} = 300 ^\circ {\text{C}}$ , $T_{red} = 500 ^\circ {\text{C}}$;

**Figure 7:**
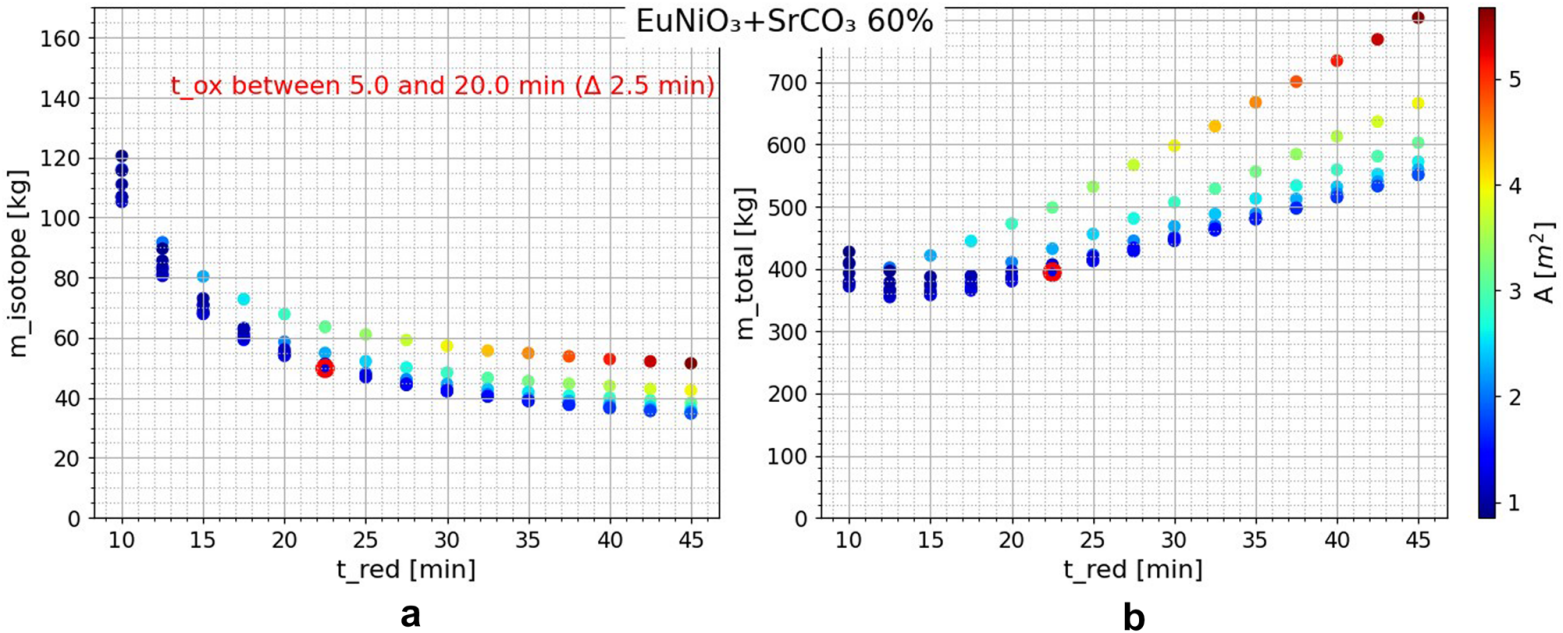
EuNiO_3_-SrCO_3_ (60% ^90^Sr) calculation results for (**a**) isotope mass $m_{{{\text{RI}}}}$, (**b**) total curtain mass $m_{{{\text{total}}}}$, (color) reactor chamber cross-sectional area $A$ over $t_{{{\text{ox}}}}$ (projected axis) and $t_{{{\text{red}}}}$ (x-axis) variation; $T_{{{\text{ox}}}} = 300\;^\circ {\text{C}}$ , $T_{{{\text{red}}}} = 425\;^\circ {\text{C}}$;

Exemplarily, the results of the combinations of EuNiO_3_-Cm_2_O_3_ (95% ^244^Cm) and EuNiO_3_-SrCO_3_ (60% ^90^Sr) are presented below in Figs. 6 and 7, respectively. By varying the upper and lower temperature, it was found that the most favorable temperature swing was between 300 and 500 °C for the former material and between 300 and 425 °C for the latter. After selecting the temperature, the times are varied and the oxidation time ($t_{{{\text{ox}}}}$) variation axis projected into the 2D plane resulted in vertical point groups of equal reduction time ($t_{{{\text{red}}}}$). Note that the best values in terms of $m_{{{\text{RI}}}}$ (and thus $\dot{Q}_{{{\text{th}}}}$) are in conflict with the equally important values of $m_{{{\text{total}}}}$ and $A$ $\left( {A \sim \dot{V}_{{{\text{ox}}}} \sim P_{{{\text{blow}}}} } \right)$. The marked cases (red circles in the diagrams) are selected as reasonably balanced cases. Tables 9 and 10 show the calculations for the chosen cases of both materials. In addition, Table 11 shows a comparison of the different isotopes in relation to the most important calculated parameters. The full corresponding data is given in the supplementary information. When comparing the different options, Cm_2_O_3_ has the lowest total mass. As already discussed, Cm_2_O_3_ emits a significant number of neutrons, which requires additional shielding. This could counteract the initial weight advantage. SrCO_3_ has the second lowest total mass and the highest thermal energy. Nevertheless, it must be noted that for the use of Sr from nuclear waste, enrichment must occur to obtain 100% ^90^Sr.

**Table 9:** Chosen case EuNiO_3_-Cm_2_O_3_ (95% ^244^Cm) calculation results; $T_{{{\text{ox}}}} = 300\;^\circ {\text{C}}$ , $T_{{{\text{red}}}} = 500\;^\circ {\text{C}}$.

$t_{{{\text{ox}}}}$[min]	$t_{{{\text{red}}}}$[min]	$m_{{{\text{ABO}}_{3} }}$[kg]	$m_{{{\text{RI}}}} *$[kg]	$m_{{{\text{RIC}}}}$[kg]	$m_{{{\text{cf}}}}$[kg]	$m_{{{\text{total}}}}$[kg]	$V_{{{\text{comp}}}}$[l]
15.0	17.5	98.66	11.07	12.16	95.8	206.62	13.83

$\dot{V}_{{{\text{ox}}}}$[m^3^/s]	$P_{{{\text{blow}}}}$[kW]	$\dot{Q}_{{{\text{ox}}}}$[kW]	$\eta_{{{\text{util}}}}$	$A$[m^2^]	$n_{{\text{p}}}$	$d_{{{\text{comp}}}}$[mm]	$\Delta T_{{{\text{CO}}_{2} }}$[K]
67.91	3.4	62.74	0.05	1.23	36.0	0.35	56.27

$*m_{{{}_{ }^{244} {\text{Cm}}\left( {95\% } \right)}}$ of $11.1 \,{\text{kg}}$ corresponds to a thermal power $(\dot{Q}_{{{\text{th}}}} )$ of $29.2 \,{\text{kW}}$.

**Table 10:** Chosen case EuNiO_3_-SrCO_3_ (60% ^90^Sr) calculation results; $T_{{{\text{ox}}}} = 300\,^\circ {\text{C}}$, $T_{{{\text{red}}}} = 425\,^\circ {\text{C}}$.

$t_{{{\text{ox}}}}$[min]	$t_{{{\text{red}}}}$[min]	$m_{{{\text{ABO}}_{3} }}$[kg]	$m_{{{\text{RI}}}} *$[kg]	$m_{{{\text{RIC}}}}$[kg]	$m_{{{\text{cf}}}}$[kg]	$m_{{{\text{total}}}}$[kg]	$V_{{{\text{comp}}}}$[l]
15.0	22.5	199.84	49.82	83.03	111.49	394.35	48.06

$\dot{V}_{{{\text{ox}}}}$[m^3^/s]	$P_{{{\text{blow}}}}$[kW]	$\dot{Q}_{{{\text{ox}}}}$[kW]	$\eta_{{{\text{util}}}}$	$A$[m^2^]	$n_{{\text{p}}}$	$d_{{{\text{comp}}}}$[mm]	$\Delta T_{{{\text{CO}}_{2} }}$[K]
78.36	3.92	68.03	0.06	1.42	39.0	1.03	52.88

$*m_{{{}_{ }^{90} {\text{Sr}}\left( {60\% } \right)}}$ of $49.8 {\text{kg}}$ corresponds to a thermal power $\left( {\dot{Q}_{{{\text{th}}}} } \right)$ of $27.5 {\text{kW}}$.

**Table 11:** Comparison of the most important calculation results for all investigated radioisotopes; for chosen balanced cases and optimized temperature swings; full corresponding data in Supplementary Information.

	Cm_2_O_3_ (95% ^244^Cm)	SrCO_3_ (100% ^90^Sr)	SrCO_3_ (60% ^90^Sr)	PuO_2_ (100% ^238^Pu)
$A$[m^2^]	1.23	1.32	1.42	1.70
$P_{{{\text{blow}}}}$[kW]	3.40	3.66	3.92	4.70
$m_{{{\text{RI}}}}$[kg]	11.07	34.62	49.82	41.02
$\dot{Q}_{{{\text{th}}}}$[kW]	29.24	31.85	27.50	23.38
$m_{{{\text{total}}}}$[kg]	206.62	285.09	394.35	338.62

### Thermal design aspects of reactor chamber

The transient thermal behavior of the reactor geometry has not been considered yet.

It is quite obvious that it is necessary to insulate the reactor chamber against the Martian ambience so that there is a minimum waste heat flow through the walls and seals.

In addition, the ‘curtains’ inside the chamber will not be cooled down uniformly because the flow boundary layers heat up towards the end/outlet of the chamber. As was mentioned previously, this is unfavorable and should be avoided if possible. Fortunately, we can solve this problem by the simple measure of making the composite layer within the curtains (i.e. $d_{{{\text{composite}}}}$) thicker towards the entrance. Thickening the layer solely while maintaining the *AB*O_3_-RIC-mixture ratio less than perfect uniformity, as the constant thickness (and thus heat capacity) of the carbon fiber fabric would interfere with the continuity. However, if this ratio is simultaneously adjusted, we can actually guarantee uniformity at the end of both steps, although for this $l$ must not be unreasonably long. Detailed simulations are necessary to know how to do this. Fabrication-wise, it is possible to add the crumbs of the two compounds accordingly and sew them into the curtains.

Another restriction arises in the optimization of the POP system due to the fact that the oxidation time ($t_{{{\text{ox}}}}$) is not truly a free variable. $t_{{{\text{ox}}}}$ determines the oxidation-end temperature $(T_{{{\text{ox}}}} )$ and therefore must be chosen accordingly. Initial CFD simulations indicate that the assumption of oxidation times of about 10–15 min is justifiable, hence the calculation carried-out above.

At this point it should be mentioned that an in-flight solution must also be found. The curtains are quite thin and since the heat production of the RIC is quite intense, the material gets too hot or it could even melt if no forced convection cools it. One possibility would be to install a thermo-oil cooling system that dissipates the decay heat to a radiator, which then radiates the heat into the space. To do this, the curtains must be brought together (which means reducing the gap width), as the entire reactor volume ($A \cdot l$) cannot be fill with thermo-oil. This has to be accomplished by an electro-mechanical mechanism.

### Dust protection

The Martian atmosphere contains considerable amounts of dust, and dust storms are very common. Therefore, dust must not be allowed to enter the POP system, especially the reactor chamber, and protective measures, e.g. against clogging and mechanical damage, must be provided; a filter may not be possible due to the large volume flow and potentially large pressure drop in the POP system. However, the proposed ‘curtain’ design is expected to provide sufficient resistance to dust particle bombardment, although clogging by dust particles can occur after many cycles. An option that is likely to result in an acceptable pressure drop is the use of a dust funnel/vortex separator, where the dust particles are propelled against the outer wall and spiral downwards through the funnel-shaped outlet by gravity.

Overview of weight and thermal performance of the POP system compared to the MOXIE system.

The weights of the various components that make up the POP system, excluding shielding, and the primary heat output are summarized in Table 12. These values are investigated for the two scenarios: (i) high ($n_{{{\text{O}}_{{2,{\text{abs}}}} }} = 0.8)$ and (ii) low ($n_{{{\text{O}}_{{2,{\text{abs}}}} }} = 0.2)$ oxygen absorption of the perovskite material. The weight of the ‘curtains’ (shown in Figs. 4 and 5) is calculated considering the weight of the carbon fiber fabrics and the enclosed granulates of perovskite radioisotope compounds. These values are estimates and need to be used with caution.

**Table 12:** Estimation of the weight of the POP system components (without shielding) and the primary thermal performance.

Component	$n_{{{\text{O}}_{2} ,{\text{abs}}}} = 0.8$		$n_{{{\text{O}}_{2} ,\,{\text{abs}}}} = 0.2$	
	Weight [kg]	Thermal energy source	Weight [kg]	Thermal energy source
Curtains	207	^244^Cm: 29.2 kW	518	^244^Cm: 73.6 kW
Reactor chamber (including seals)	110		300	
Reactor chamber insulation	50		120	
Thermoelectric modules	50		100	
Support structure (E.G. standing feet)	150		300	
Electric blower	100		200	
Electric motor	20		30	
Electric storage	50		125	
Liquefaction	70		70	
5 kw_e_ KRUSTY reactor	1000	HEU*: 21.5 kW	1500**	HEU: 43 kW**
Total	1807 kg	50.7 kW	3263 kg	116.6 kW

*Highly enriched uranium (HEU)^55^.

**10 kw_e_ KRUSTY Reactor.

It is important to note that the actual percentage of oxygen uptake depends on a number of factors, including the material-specific oxidation kinetics, the structure of the POP system and the operating conditions, which need to be optimized in an experimental study. However, it is clear from the literature that oxygen uptake in perovskites is enhanced when a lower oxygen content is present in the gas stream^54^. Therefore, with optimized operating conditions, high oxygen uptake is more likely.

One option, if the excess heat from the reaction chamber is sufficient, is a 5 kW_e_ KRUSTY-fission reactor, which even enables power distribution to the liquefaction stream demand and a short-term storage solution.

Additional power could be generated by partially replacing the reactor chamber insulation with e.g. thermoelectric modules and adjusting the RIC quantity and distribution inside, resulting in a reactor chamber RTG. Other options for power generation are a dedicated external RTG/Advanced Stirling Radioisotope Generator (ASRG) and solar solutions. The weight of the CO_2_ electrolysis ISRU option is significantly higher in comparison due to the high demand for thermal energy to produce approximately 3 kg of O_2_ per hour^56^ while in operation, as illustrated in Table 13.

**Table 13:** Estimation of the weight of the MOXIE system components and the thermal performance.

Component	Weight [kg]	Thermal energy source
Upscaled MOXIE + Liquefaction	1000**	
Three 10 kW_e_ KRUSTY-Reactors	3 1500	HEU: 3 43 kW*
Total	5500 kg	129 kW

*^55^.

**^57^.

## Conclusions and outlook

It is shown that the concentration of oxygen by thermochemical processes in the extraterrestrial atmosphere is feasible, and a suitable best-case design for compact apparatus and corresponding considerations are investigated. For the radioactive materials of the POP system, ^244^Cm proves to be the best option from the point of view of gravimetric energy density. However, it is unlikely to be used because of the high shielding requirements^52^. On the other hand, the procurement of 40 kg of ^238^Pu is impossible due to the current supply shortages. Hence, ^90^Sr is the most reasonable option, but if it is not possible to purify the strontium content of the nuclear waste (in form of ^90^Sr), the POP system loses much of its suitability compared to nuclear fission. Nevertheless, there is still the possibility of supplying the required heat via an external source, e.g. a fission reactor and a heat transport fluid (e.g. nitrate salt). In this approach, the primary thermal energy is directly used and no additional conversion to electricity is needed. Therefore, a higher energy efficiency and a reduction of the total heat waste compared to the MOXIE approach is expected. However, a particular challenge with this alternative approach is that the heat transfer fluid can flow through the perovskite geometry of the reactor chamber without making it much heavier, as such an increase in size would require more heat. Radiation shielding still needs to be investigated in more detail, as it is likely to have a significant impact on the overall weight. Although there are still some uncertainties, significant weight savings seem possible compared to other ISRU systems, even considering the most unfavorable conditions and the maximum weight requirement to obtain 2.25 kg of oxygen per hour. In a Mars mission, as in spaceflight in general, the payload has a major impact on the cost of the mission. Even a relatively small amount of further research should be able to answer the question of whether a POP system is indeed viable in all its aspects. Considering the general cost of an extraterrestrial mission and the potential savings in the case of a well-designed and functioning POP system, such efforts are a very small price to pay for a potentially very large gain.

Moreover, this work revealed that various aspects of the POP system are complex and interconnected and therefore require further theoretical and experimental investigation. In addition, there are some weaknesses and uncertainties to be considered in future work: (i) The non-stoichiometric equilibrium data for the perovskites (including EuNiO_3_) were calculated theoretically using estimates, so experimental validation is still required; (ii) It will be investigated by means of phase diagram and experimental validation that no reaction occurs between the redox material and the RIC; (iii) A transient thermal simulation of the heat distribution during operation and flight is needed for the proposed fabric-like carbon fiber ‘curtains’, and thus experimental results or a detailed (thermo-)mechanical calculation of the actual ‘curtains’ design must ensure the stability of the fabric-like pattern, as the estimated pressure drop is based on a simplified CFD simulation and the values are therefore unsubstantiated; (iv) The kinetics of perovskite gas exchange, i.e. the oxygen uptake of the curtains from the flow and their oxygen release during reduction, needs to be investigated experimentally; (v) Evaluation and validation of the proposed and/or alternative adequate protection solutions against Mars dust of different particle sizes, as a filter may not be suitable due to the large volume flow and potentially large pressure drop.

Experimental analyses for specific reactor geometries (including ‘curtains’ gap width, reactor chamber length, heating rate, turbulence flow, etc.) and design stability for the POP system need to be carried-out, as well as a detailed analysis of radiation protection and shielding requirements and the actual weight of the required shielding. In addition, the effect of carbonization during oxidation on the performance of the POP system needs to be investigated, and waste heat utilization and alternative heat sources such as fission reactor need to be evaluated and the new reactor chamber geometry reconsidered to allow for the modifications.

## Supplementary Information

Supplementary Information.

## Data Availability

The datasets used and/or analysed during the current study available from the corresponding author on reasonable request.

## References

[1] Jackson, G. S., Imponenti, L., Albrecht, K. J., Miller, D. C. & Braun, R. J. Inert and reactive oxide particles for high-temperature thermal energy capture and storage for concentrating solar power. *J. Sol. Energy Eng.* **141**(2), 021016 (2019).

[2] Hu J, Hongmanorom P, Galvita VV, Li Z, Kawi S (2021). Bifunctional Ni-Ca based material for integrated CO_2_ capture and conversion via calcium-looping dry reforming. Appl. Catal. B.

[3] Yilmaz D, Darwish E, Leion H (2021). Utilization of promising calcium manganite oxygen carriers for potential thermochemical energy storage application. Ind. Eng. Chem. Res..

[4] Buelens LC, Poelman H, Marin GB, Galvita VV (2019). 110th anniversary: carbon dioxide and chemical looping: Current research trends. Ind. Eng. Chem. Res..

[5] Kim SM (2018). Integrated CO_2_ capture and conversion as an efficient process for fuels from greenhouse gases. ACS Catal..

[6] Antzaras AN, Heracleous E, Lemonidou AA (2021). Hybrid catalytic materials with CO2 capture and oxygen transfer functionalities for high–purity H2 production. Catal. Today.

[7] Pröll, T. in *Calcium and Chemical Looping Technology for Power Generation and Carbon Dioxide (CO2) Capture* 197–219 (Elsevier, 2015).

[8] Buck, R., Agrafiotis, C., Tescari, S., Neumann, N. & Schmücker, M. Techno-economic analysis of candidate oxide materials for thermochemical storage in concentrating solar power systems. *Front. Energy Res.* **9**, 322 (2021).

[9] Vieten J (2016). Perovskite oxides for application in thermochemical air separation and oxygen storage. J. Mater. Chem. A.

[10] Bulfin B, Vieten J, Agrafiotis C, Roeb M, Sattler C (2017). Applications and limitations of two step metal oxide thermochemical redox cycles: A review. J. Mater. Chem. A.

[11] Bulfin B (2017). Redox chemistry of CaMnO_3_ and Ca_0.8_Sr_0.2_MnO_3_ oxygen storage perovskites. J. Mater. Chem. A.

[12] Bulfin B (2019). Air separation and selective oxygen pumping via temperature and pressure swing oxygen adsorption using a redox cycle of SrFeO3 perovskite. Chem. Eng. Sci..

[13] Pein M (2020). Redox thermochemistry of Ca–Mn-based perovskites for oxygen atmosphere control in solar-thermochemical processes. Sol. Energy.

[14] Vieten J (2019). Redox behavior of solid solutions in the SrFe1-xCuxO3-δ system for application in thermochemical oxygen storage and air separation. Energy Technol. Ger..

[15] Fennell, P. & Anthony, B. *Calcium and Chemical Looping Technology for Power Generation and Carbon Dioxide (CO*_*2*_*) Capture* (Elsevier, 2015).

[16] Liu X, Zhang H, Hong H, Jin H (2020). Experimental study on honeycomb reactor using methane via chemical looping cycle for solar syngas. Appl. Energ.

[17] Rinehart GH (2001). Design characteristics and fabrication of radioisotope heat sources for space missions. Prog. Nucl. Energy.

[18] Ritz, F. & Peterson, C. E. in *2004 IEEE Aerospace Conference Proceedings (IEEE Cat. No.04TH8720)* 2950–2957 (IEEE, 2004).

[19] Matthes, C. S. R. *et al. *in* 2018 IEEE Aerospace Conference* 1–9 (IEEE, 2018).

[20] Werner, J., Lively, K. & Kirkham, D. in *2017 IEEE Aerospace Conference* 1–6 (IEEE, 2017).

[21] Jiang, M. An overview of radioisotope thermoelectric generators. *Introduction to Nuclear Energy PH241-Stanford University-Winter* (2013).

[22] Vieten J (2019). Materials design of perovskite solid solutions for thermochemical applications. Energy Environ. Sci..

[23] Klaas, L., Kriechbaumer, D., Roeb, M. & Vieten, J. Thermochemisches Verfahren und kompakte Apparatur zur Aufkonzentration von Sauerstoff in extraterrestrischen Atmosphären. DE 10 2021 121 911 A1 (2019).

[24] Bulfin, B. *et al.* Isothermal relaxation kinetics for the reduction and oxidation of SrFeO_3_ based perovskites. *Phys. Chem. Chem. Phys.* **22**(4), 2466–2474 (2020). 10.1039/c9cp05771d 31939962

[25] Vieten, J., Kriechbaumer, D., Klaas, L. & Roeb, M. *Thermochemisches Verfahren und kompakte Apparatur zur Aufkonzentration von Sauerstoff in extraterrestrischen Atmosphären* (2021).

[26] Trainer MG (2019). Seasonal variations in atmospheric composition as measured in gale crater. Mars. J. Geophys. Res. Planets.

[27] Jet Propulsion Laboratory, California Institute of Technology, MARSTrek-NASA. https://trek.nasa.gov/mars/ (2022).

[28] Harrison DP (2008). Sorption-enhanced hydrogen production: a review. Ind. Eng. Chem. Res..

[29] Golombek et al. SPACEX STARSHIP LANDING SITES ON MARS. Available at https://www.hou.usra.edu/meetings/lpsc2021/search/?q=site%3Awww.hou.usra.edu%2Fmeetings%2Flpsc2021%2F&cx=8f94017e0be096968&cof=FORID%3A11&q=spacex+landing&sa=Search (2021).

[30] Golombek, M. et al. *SPACEX STARSHIP LANDING SITES ON MARS*. 52nd Lunar and Planetary Science Conference, LPI Contrib. No. 2548 (2021).

[31] Martínez GM (2017). The modern near-surface martian climate: A review of in-situ meteorological data from viking to curiosity. Space Sci. Rev..

[32] Forget F (1999). Improved general circulation models of the Martian atmosphere from the surface to above 80 km. J. Geophys. Res. Planets.

[33] Millour, E. *et al*. *The Mars Climate Database (version 5.3). Scientific Workshop “from Mars express to ExoMars”* (2018).

[34] Brendelberger S, von Storch H, Bulfin B, Sattler C (2017). Vacuum pumping options for application in solar thermochemical redox cycles–Assessment of mechanical-, jet-and thermochemical pumping systems. Sol. Energy.

[35] Zhang Z, Ou Z, Qin C, Ran J, Wu C (2019). Roles of alkali/alkaline earth metals in steam reforming of biomass tar for hydrogen production over perovskite supported Ni catalysts. Fuel.

[36] Afzal S, Sengupta D, Sarkar A, El-Halwagi M, Elbashir N (2018). Optimization approach to the reduction of CO_2_ emissions for syngas production involving dry reforming. ACS Sustain. Chem. Eng..

[37] Rönsch S (2016). Review on methanation–from fundamentals to current projects. Fuel.

[38] Schmücker, M. & Schneider, H. in *Handbook of Ceramic Composites* (ed Bansal, N. P.) 423–435 (Kluwer Academic Publishers, 2005).

[39] Schmücker M, Grafmüller A, Schneider H (2003). Mesostructure of WHIPOX all oxide CMCs. Compos. A Appl. Sci. Manuf..

[40] Gnielinski V (2010). VDI Heat Atlas.

[41] Angeli SD, Monteleone G, Giaconia A, Lemonidou A (2014). State-of-the-art catalysts for CH_4_ steam reforming at low temperature. Int. J. Hydrog. Energy.

[42] van Velzen D (1980). Development and design of a continuous laboratory-scale plant for hydrogen production by the Mark-13 cycle. Int. J. Hydrog. Energ.

[43] Johnson WL (2018). Comparison of oxygen liquefaction methods for use on the Martian surface. Cryogenics.

[44] Vieten, J. *Perovskite Materials Design for Two-step SolarThermochemical Redox Cycles*. A Ph.D. Dissertation. (Technische Universität Dresden, Germany, 2019).

[45] Glasner A, Levy E, Steinberg M (1963). Thermal decomposition of europium(III) oxalate. J. Inorg. Nucl. Chem..

[46] Shaheen W (2002). Thermal behaviour of pure and binary basic nickel carbonate and ammonium molybdate systems. Mater. Lett..

[47] Chen C, Yang C, Ranjan D, Loutzenhiser PG, Zhang ZM (2020). Spectral radiative properties of ceramic particles for concentrated solar thermal energy storage applications. Int. J. Thermophys..

[48] Howe, S. D., Crawford, D., Navarro, J. & Ring, T. Economical Production of Pu-238. In *Proc. of Nuclear and Emerging Technologies for Space*. Paper 6700 (2013).

[49] von Storch, H. *Methanol production via solar reforming of methane = Methanolherstellung duch solare Reformierung von Methan*. A Ph.D. Dissertation. (RWTH Aachen, Germany, 2016).

[50] Omodolor IS, Otor HO, Andonegui JA, Allen BJ, Alba-Rubio AC (2020). Dual-function materials for CO_2_ capture and conversion: A review. Ind. Eng. Chem. Res..

[51] Brémond U, Bertrandias A, Steyer J-P, Bernet N, Carrere H (2021). A vision of European biogas sector development towards 2030: Trends and challenges. J. Clean. Prod..

[52] Tian S, Yan F, Zhang Z, Jiang J (2019). Calcium-looping reforming of methane realizes in situ CO_2_ utilization with improved energy efficiency. Sci. Adv..

[53] Halmann M, Steinfeld A (2003). Thermoneutral coproduction of calcium oxide and syngas by combined decomposition of calcium carbonate and partial oxidation/CO_2_-reforming of methane. Energy Fuels.

[54] Capstick S, Bulfin B, Naik JM, Gigantino M, Steinfeld A (2023). Oxygen separation via chemical looping of the perovskite oxide Sr_0.8_Ca_0.2_FeO_3_ in packed bed reactors for the production of nitrogen from air. Chem. Eng. J..

[55] Lyon RK, Cole JA (2000). Unmixed combustion: An alternative to fire. Combust. Flame.

[56] Hinterman, E., Carroll, K., Nikicio, A., de Weck, O. & Hoffman, J. In *2021 IEEE Aerospace Conference (50100)* (2021).

[57] Ackerman, E. MOXIE Might Be the Most Exciting Thing Perseverance Has Brought to Mars. Available at https://spectrum.ieee.org/moxie-might-be-the-most-exciting-thing-perseverance-has-brought-to-mars (2021).

