# Supplementary material for: Thermochemical process and compact apparatus for concentrating oxygen in extraterrestrial atmospheres: a feasibility study
Source: Sci Rep. 2023 Mar 29;13:5148. doi: 10.1038/s41598-023-31120-x (PMC10060405; doi:10.1038/s41598-023-31120-x)
Supplement: Supplementary file 1 — Supplementary Information. [file 41598_2023_31120_MOESM1_ESM.docx]

Supplementary Information

**Thermochemical Process and Compact Apparatus for Concentrating Oxygen in Extraterrestrial Atmospheres: A Feasibility Study**

*Asmaa Eltayeb^a,*^, Lena Klaas^a,c,*^, Leonhard Kölz^a^, Josua Vieten^a,b^, Martin Roeb^a^, Christian Sattler^a,c^*

^a^Institute of Future Fuels, Deutsches Zentrum
für Luft- und Raumfahrt/German Aerospace Center (DLR) Linder Höhe, 51147 Köln, Germany.

^b^ExoMatter GmbH, Leutstettener Str. 67, 81477 Munich, Germany.

^c^ RWTH Aachen University, Faculty of Mechanical Engineering, Chair for Solar Fuel

Production, 52062 Aachen, Germany.

*Corresponding authors: e-mail: [asmaa.eltayeb@dlr.de](mailto:asmaa.eltayeb@dlr.de), lena.klaas@dlr.de

# MATERIAL SELECTION

The equilibrium curves for the promising perovskite materials EuCuO_3_, LaAgO_3_, Sm_0.5_La_0.5_CoO_3_, and Sm_0.5_La_0.5_NiO_3_, selected from the list of perovskite materials suitable for two-stage thermochemical cycling by Vieten et al. ^1,2^ and investigated theoretically in this study.


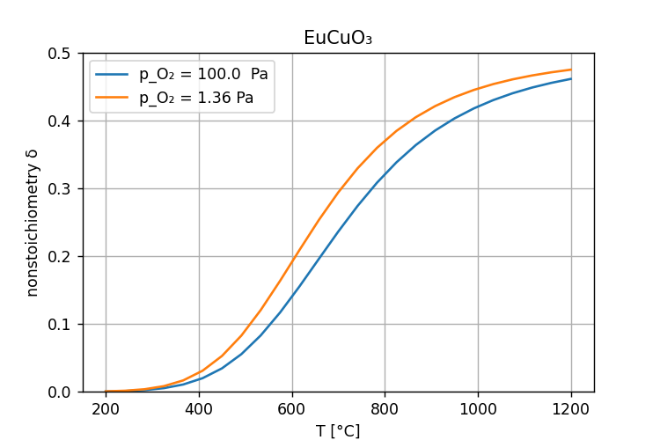

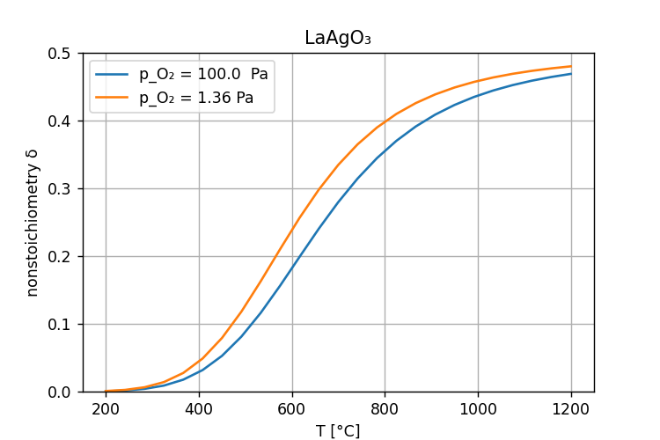

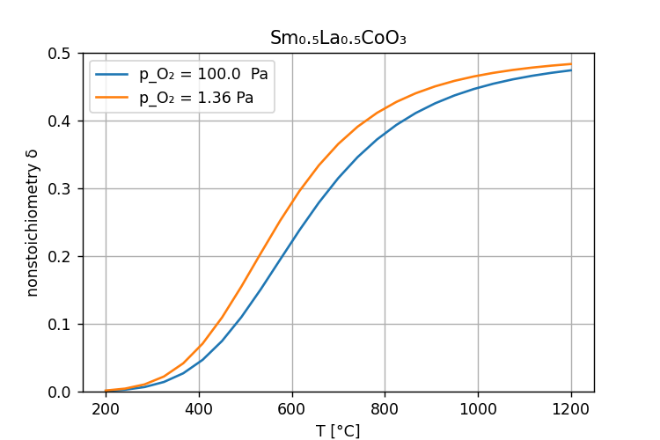

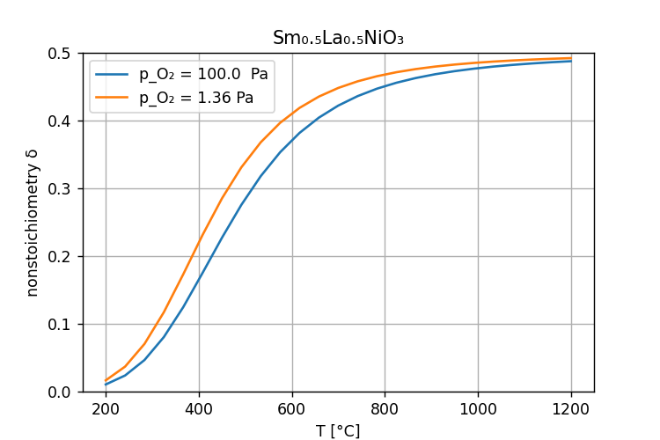


Figure 1: Two non-stoichiometry equilibrium curves of the perovskites: EuCuO_3_, LaAgO_3_, Sm_0.5_La_0.5_CoO_3_, and Sm_0.5_La_0.5_NiO_3_.

# MATERIAL PROPERTIES

## EuNiO3

Enthalpy: $\Delta H_{O, EuNiO_{3}}=74 \frac{\mathrm{kJ}}{\mathrm{mol}}$ ^3^

Molecular Mass: $M_{\mathrm{EuNi}O_{3}}=258.7\frac{g}{\mathrm{mol}}$

Density: $\rho_{\mathrm{EuNi}O_{3}}=7.8\frac{g}{\mathrm{cm}^{3}}$ ^4^

Molar Heat Capacity: $c_{p,EuNiO_{3}}=125\frac{J}{mol\cdot K}$ ^5^*
* Debye model for perovskite

## Radioisotopes

Table 1: Properties of Common Radioisotopes (RI).

| **Radioisotope** | **^238^Pu** | **^90^Sr** | **^244^Cm** |
| --- | --- | --- | --- |
| Molar weight  $M_{\mathrm{RI}}\mathrm{in}\left[ g/\mathrm{mol} \right]$ | **238** | **90** | **244** |
| Half time $t_{1/2,\mathrm{RI}} in [y]$ | **87.7**  ^6^ | **28.9**  ^6^ | **18.1**  ^6^ |
| Mean heat per decay  $\bar{Q}_{decay,RI} in [MeV]$ | **5.593**  ^6^ | **1.128 ^*a^**  ^6^ | **5.795**  ^6^ |
| Weight specific thermal power  $\dot{q}_{\mathrm{RI}} \mathrm{in}\left[ W/g \right]$ | **0.568** | **0.919** | **2.782** |
| **Corresponding radioisotope-compound** | **PuO_2_** | **SrCO_3_** | **Cm_2_O_3_** |
| Molar weight  $M_{\mathrm{RIC}}\mathrm{in}\left[ g/\mathrm{mol} \right]$ | **270** | **150** | **536** |
| Weight specific thermal power for 100% active RI  $\dot{q}_{\mathrm{RIC}}\mathrm{in}\left[ W/g \right]$ | **0.5** | **0.55** | **2.53** |
| Volume specific thermal power  $\dot{q}_{\mathrm{RIC}} \mathrm{in}\left[ W/{\mathrm{cm}^{3}} \right]$ | **5.1** | **2.0** | **26.1** |
| Density  $\rho_{\mathrm{RIC}}\mathrm{in}\left[ g/{\mathrm{cm}^{3}} \right]$ | **10.2**  ^7^ | **3.7**  ^8^ | **10.3**  ^9^ |
| Specific molar heat capacity at 400°C  $c_{p,RIC}\mathrm{in}\left[ J/{mol/K} \right]$ | **86**  ^10^ | **111**  ^11^ | **130**  ^12^ |

*a: $\bar{Q}_{decay,{}^{90}\mathrm{Sr}}=\bar{Q}_{decay,{}^{90}\mathrm{Sr}+{}^{90}Y}=\left( 0.196+0.932 \right)\mathrm{MeV}= 1.128 \mathrm{MeV}$

**The mass-specific decay heat power of radioisotopes; calculations:**

The radioisotopes mass-specific decay heat was calculated using the raw data for the mean energy per decay $(\bar{Q}_{\mathrm{decay}})$ and the half time $(t_{1/2})$ from [1] according to

$$a_{\mathrm{RI}}=\frac{0.693\cdot6.022e23\frac{1}{\mathrm{mol}}}{t_{1/2}\cdot365\cdot24\cdot3600s} in \left[ Bq/mol \right], t_{1/2} in \left[ a \right],$$

$$\tilde{\dot{q}_{\mathrm{RI}}}=\bar{Q}_{\mathrm{decay}}\cdot a_{\mathrm{RI}}\cdot1.602e-13\frac{J}{\mathrm{MeV}} in \left[ W/\mathrm{mol} \right], \bar{Q}_{\mathrm{decay}} in [\mathrm{MeV}]$$

and

$$\dot{q}_{\mathrm{RI}}=\frac{\tilde{\dot{q}_{\mathrm{RI}}}}{M_{\mathrm{RI}}} in [W/g]$$

## Carbon Fibers

In ^13^, a density ${(\rho}_{\mathrm{cf}})$ of carbon fibers of 1.76 g/cm^3^, a specific heat capacity ($c_{p,cf})$ of 0.78 J/(g∙K), a tensile strength of 3.5 GPa and a tensile modulus of 230 GPa are given.

In ^14^ it is shown that by producing hollow fibers using different production techniques, the density can be lowered to obtain a fiber that has a density of 1.20 g/cm^3^, a tensile strength of 1.6 GPa and a tensile modulus of 244 GPa.

It can therefore reasonably be assumed that such hollow fibers have a similar mass-specific heat capacity. Thus, the following properties are used for carbon fibers:

$$\rho_{\mathrm{cf}}=1.2g/{{cm}^{2}}, c_{p,cf}=0.78J/{g\cdot K}$$

The influence of the Al_2_O_3_ coating on weight and heat capacity is neglected because the layer is very thin compared to the thickness of a single fiber ^15^.

We decided on a thickness of 1 mm for the “plates”, which results in 1200 g/m^2^ (1200 gsm). Whether this results in a sufficiently stable and durable enough carbon fiber fabric remains to be investigated. An example of carbon fibre fabric can be seen in Fig. 1.


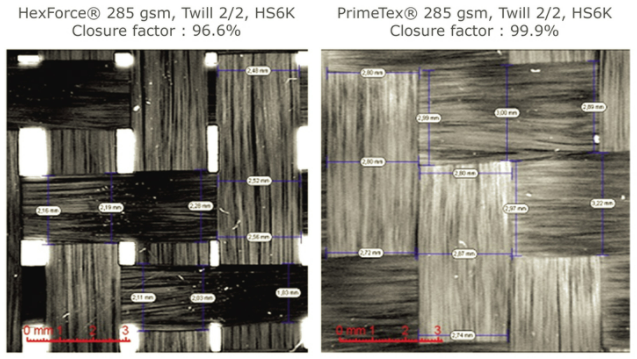


Figure 2: Hexcel Hexforce carbon fiber fabric ^16^

## Python Code Equations

The equations used are listed in the same order as in the python code.

The mean volume flow $\dot{(V})$ to be pumped through the apparatus, assuming ideal gas and a proportion of unabsorbed oxygen $(\varepsilon_{O_{2}})$ remaining in the exhaust stream:

$$\begin{aligned} \dot{V}=\dot{V}_{O_{2}}=\frac{\dot{m}_{O_{2}}\cdot\frac{R}{M_{O_{2}}}\cdot T_{M}}{p_{O_{2},M}}\cdot\frac{1}{1-\varepsilon_{O_{2}}} \left[ \frac{m^{3}}{h} \right]\#( SEQ Formel \backslash* ARABIC 1) \end{aligned}$$

The mean blower power $\bar{(P}_{\mathrm{blow}})$:

$$\begin{aligned} \bar{P}_{\mathrm{blow}}=\frac{\dot{V}\cdot\Delta p}{3600} \left[ W \right]\#\left( SEQ Formel \backslash* ARABIC 2 \right) \end{aligned}$$

**Number of oxidation-reduction cycles per hour** ${(n}_{cycle})$**:**

$$\begin{aligned} n_{cycle}=\frac{1}{t_{\mathrm{ox}}+t_{\mathrm{red}}} \left[ \frac{1}{h} \right] \#\left( SEQ Formel \backslash* ARABIC 3 \right) \end{aligned}$$

Oxygen quantity per cycle:

$$\begin{aligned} m_{O_{2}}=\frac{\dot{m}_{O_{2}}}{cyc} [g]\#\left( SEQ Formel \backslash* ARABIC 4 \right) \end{aligned}$$

$$\begin{aligned} n_{O_{2}}=\frac{m_{O_{2}}}{M_{O_{2}}} [mol]\#\left( SEQ Formel \backslash* ARABIC 5 \right) \end{aligned}$$

Perovskite quantity ${(n}_{\mathrm{AB}O_{3}})$:

$$\begin{aligned} n_{\mathrm{AB}O_{3}}=n_{O_{2}}\cdot\frac{2}{\Delta\delta}\left( =\frac{\dot{m}_{O_{2}}}{cyc\cdot M_{O_{2}}}\cdot\frac{2}{\Delta\delta}=\frac{\dot{m}_{O_{2}}}{\frac{1}{t_{\mathrm{ox}}+t_{\mathrm{red}}}\cdot M_{O_{2}}}\cdot\frac{2}{\Delta\delta} \right) [mol]\#\left( SEQ Formel \backslash* ARABIC 6 \right) \end{aligned}$$

$$\begin{aligned} m_{\mathrm{AB}O_{3}}=n_{\mathrm{AB}O_{3}}\cdot M_{\mathrm{AB}O_{3}} [g]\#\left( SEQ Formel \backslash* ARABIC 7 \right) \end{aligned}$$

Volume flow during oxidation $(\dot{V}_{\mathrm{ox}})$:

$$\begin{aligned} \dot{V}_{\mathrm{ox}}=\frac{\dot{V}}{cyc\cdot t_{\mathrm{ox}}} \left[ \frac{m^{3}}{h} \right] \#\left( SEQ Formel \backslash* ARABIC 8 \right) \end{aligned}$$

Blower power ${(P}_{\mathrm{blow}})$:

$$\begin{aligned} P_{\mathrm{blow}}=\frac{\dot{V}_{\mathrm{ox}}\cdot\Delta p}{3600} \left[ W \right]\#\left( SEQ Formel \backslash* ARABIC 9 \right) \end{aligned}$$

Speed of sound in carbon dioxide ambient $(c_{\mathrm{CO}_{2}})$:

$$\begin{aligned} c_{\mathrm{CO}_{2}}=\sqrt{\kappa\cdot\frac{R\cdot T}{M_{\mathrm{CO}_{2}}}} \left[ \frac{m}{s} \right]\#\left( SEQ Formel \backslash* ARABIC 10 \right) \end{aligned}$$

Reactor chamber inlet flow velocity$(v_{0})$:

$$\begin{aligned} v_{0}=0.25\cdot c_{\mathrm{CO}_{2}} \left[ \frac{m}{s} \right] \#\left( SEQ Formel \backslash* ARABIC 11 \right) \end{aligned}$$

Reactor chamber cross-sectional area $(A)$:

$$\begin{aligned} A=\frac{\dot{V}_{\mathrm{ox}}}{v_{0}\cdot3600} {[m}^{2}]\#\left( SEQ Formel \backslash* ARABIC 12 \right) \end{aligned}$$

Plate/curtain number ${(n}_{p})$:

$$\begin{aligned} n_{p}\approx\frac{\sqrt{A}}{s_{p}} \#\left( SEQ Formel \backslash* ARABIC 13 \right) \end{aligned}$$

Carbon fiber mass ${(m}_{\mathrm{cf}})$:
Under the simplification that the woven fabric is a plate with a certain thickness.

$$\begin{aligned} m_{\mathrm{cf}}=n_{p}\cdot\left( l\cdot\sqrt{A}\cdot d_{p} \right)\cdot\left( \rho_{\mathrm{cf}}\cdot1e6 \right) \left[ g \right]\#\left( SEQ Formel \backslash* ARABIC 14 \right) \end{aligned}$$

Minimum reduction time:
An increase in heat flux means an increase in isotope-compound-mass, which also means an increase in the mass to-be-heated up and thus the heat capacity. A reduction time below $t_{red,min}$ means that the latter surpasses the former.

$$\begin{aligned} t_{red,min}=\frac{c_{p, RIC}\cdot\left( T_{2}-T_{1} \right)}{3600\cdot M_{\mathrm{ri}}\cdot\dot{q}_{\mathrm{ri}}} [h]\#\left( SEQ Formel \backslash* ARABIC 15 \right) \end{aligned}$$

The code loops the following equations (15) to (19) because eq. (15) = *f* ($n_{\mathrm{RIC}}$).

Energy quantity to heat up the entire plate/curtain mass per cycle $(\Delta Q_{m})$:

$$\begin{aligned} \Delta Q_{m}=\left( \left( c\cdot n \right)_{\mathrm{RIC}}+\left( c\cdot n \right)_{\mathrm{AB}O_{3}}+\left( c\cdot m \right)_{\mathrm{cf}} \right)\cdot\left( T_{2}-T_{1} \right)\cdot\frac{1}{3600} [Wh]\#\left( SEQ Formel \backslash* ARABIC 16 \right) \end{aligned}$$

Reaction heat per cycle $(\Delta Q_{r})$:

$$\begin{aligned} \Delta Q_{r}=\Delta H_{O,ABO_{3}}\cdot n_{\mathrm{AB}O_{3}}\cdot\Delta\delta\cdot\frac{1}{3600} [Wh]\#\left( SEQ Formel \backslash* ARABIC 17 \right) \end{aligned}$$

Thermal power $(\dot{Q}_{\mathrm{th}})$:

The thermal power is only applicable during reduction time.

$$\begin{aligned} \dot{Q}_{\mathrm{th}}=\frac{\left. \Delta Q_{m}+\Delta Q_{r} \right.}{t_{\mathrm{red}}} [W]\#\left( SEQ Formel \backslash* ARABIC 18 \right) \end{aligned}$$

Radioisotope mass${(m}_{\mathrm{RI}})$:

$$\begin{aligned} m_{\mathrm{RI}}=\frac{\dot{Q}_{\mathrm{th}}}{\dot{q}_{\mathrm{RIi}}} [g]\#\left( SEQ Formel \backslash* ARABIC 19 \right) \end{aligned}$$

Radioisotope-compound (RIC) quantity $(n_{\mathrm{RIC}})$:

$$\begin{aligned} n_{\mathrm{RIC}}=\frac{m_{\mathrm{RI}}}{M_{\mathrm{RI}}}\cdot\frac{1}{n_{\frac{RI}{RIC}}} [mol]\#\left( SEQ Formel \backslash* ARABIC 20 \right) \end{aligned}$$

$$\begin{aligned} m_{\mathrm{RIC}}=n_{\mathrm{RIC}}\cdot M_{\mathrm{RIC}} \#\left( SEQ Formel \backslash* ARABIC 21 \right) \end{aligned}$$

where $n_{\frac{RI}{RIC}}$ is the quantity of RI per RIC molecule.

Mean heat power $\dot{(Q}_{\mathrm{ox}})$ carried away from the blown stream during oxidation:

Assuming a 10% lower reaction heat due to entropy change and the temperature-dependent redox enthalpy.

$$\begin{aligned} \dot{Q}_{\mathrm{ox}}=\dot{Q}_{\mathrm{th}}+\frac{\Delta Q_{m}+\Delta Q_{r}\cdot0.9}{t_{\mathrm{ox}}} [W]\#\left( SEQ Formel \backslash* ARABIC 22 \right) \end{aligned}$$

Mean temperature difference ${(\Delta T}_{\mathrm{CO}_{2}})$of blown stream between reactor chamber inlet and outlet:

Assuming the ideal gas law and an atmosphere of 100% CO_2_.

$$\begin{aligned} {\Delta T}_{\mathrm{CO}_{2}}=\frac{\dot{Q}_{\mathrm{ox}}}{\dot{m}_{\mathrm{CO}_{2}}\cdot c_{p,\mathrm{CO}_{2}}}=\frac{\dot{Q}_{\mathrm{ox}}}{\frac{\dot{V}_{\mathrm{ox}}\cdot p_{M}}{R\cdot T_{M}}\cdot M_{\mathrm{CO}_{2}}\cdot c_{p,\mathrm{CO}_{2}}} [K]\#\left( SEQ Formel \backslash* ARABIC 23 \right) \end{aligned}$$

Volume of RIC-ABO_3_-composite $(V_{\mathrm{comp}})$:

$$\begin{aligned} V_{\mathrm{comp}}=\frac{m_{\mathrm{RIC}}}{\rho_{\mathrm{RIC}}}+\frac{m_{\mathrm{AB}O_{3}}}{\rho_{\mathrm{AB}O_{3}}} \left[ m^{3} \right]\#\left( SEQ Formel \backslash* ARABIC 24 \right) \end{aligned}$$

Thickness of composite layer ${(d}_{\mathrm{comp}})$:

$$\begin{aligned} d_{\mathrm{comp}}=\frac{V_{\mathrm{comp}}}{l\cdot h\cdot n_{p}} [m]\#\left( SEQ Formel \backslash* ARABIC 25 \right) \end{aligned}$$

Theoretical thickness for block-like density. The actual thickness is higher due to gaps and voids between the crumbs.

Efficiency${(\eta}_{\mathrm{util}})$ that would be required to power the electric blower when utilizing the waste heat: $\begin{aligned} \eta_{\mathrm{util}}=\frac{P_{\mathrm{blow}}}{\dot{Q}_{\mathrm{ox}}} \#\left( SEQ Formel \backslash* ARABIC 26 \right) \end{aligned}$

# OPTIMIZATION RESULTS

In this section, two conditions are assumed for oxygen absorption from the input stream through the perovskite material: (i) 80% ($n_{O_{2, abs}}=0.8)$ and (ii) 20% ($n_{O_{2, abs}}=0.2)$.

## FOR 80% OXYGEN ABSORPTION $\boldsymbol{(}\boldsymbol{n}_{\mathbf{O}_{\mathbf{2, abs}}}\boldsymbol{=0.8}$)

## EuNiO_3_-Cm_2_O_3_ (95% ^244^Cm)


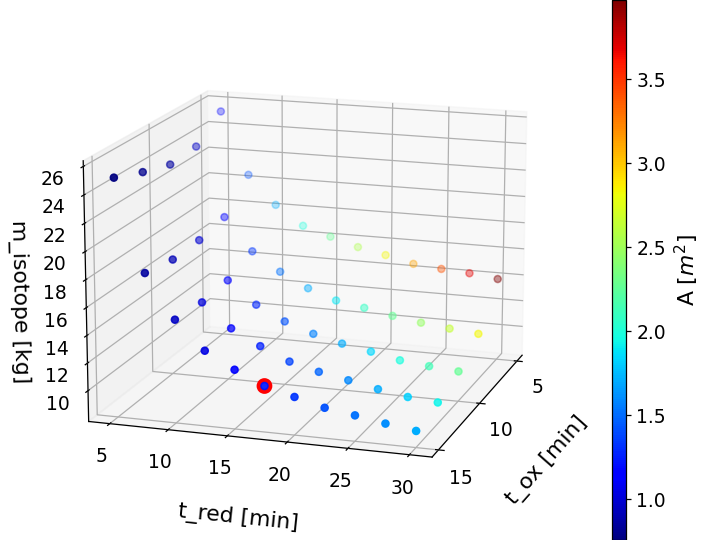

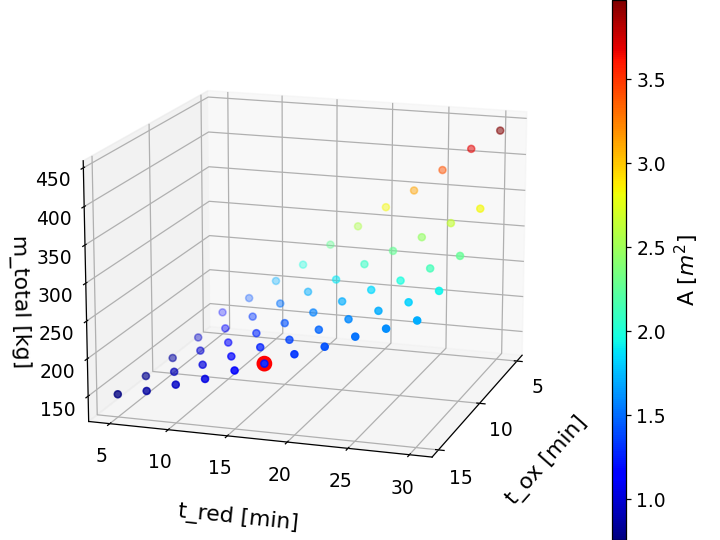


Figure 3: 3D EuNiO_3_-Cm_2_O_3_ (95% ^244^Cm) calculation results over $t_{\mathrm{ox}}$ and $t_{\mathrm{red}}$; $T_{1}=300 ^{\circ}C$ , $T_{2}=500 ^{\circ}C$; (a) $m_{\mathrm{RI}}$ (b) $m_{\mathrm{total}}$

## EuNiO_3_-SrCO_3_ (100% ^90^Sr):


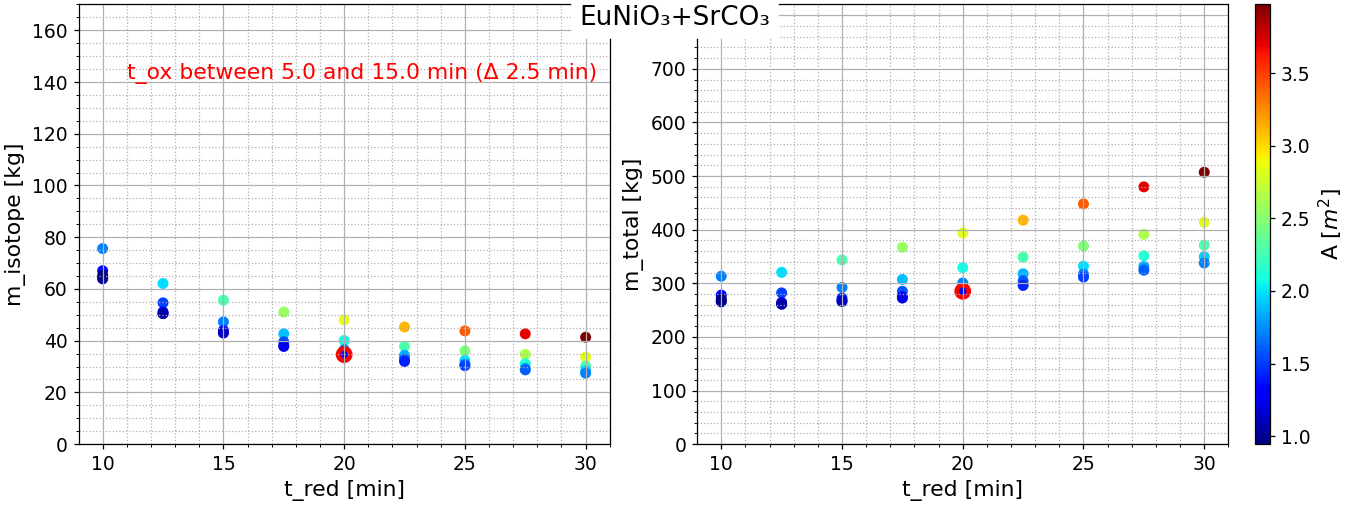


Figure 4: 2D EuNiO_3_-SrCO_3_ (100% ^90^Sr) calculation results over $t_{\mathrm{ox}}$ and $t_{\mathrm{red}}$; $T_{1}=300 ^{\circ}C$ , $T_{2}=475 ^{\circ}C$; (a) $m_{\mathrm{RI}}$ (b) $m_{\mathrm{total}}$


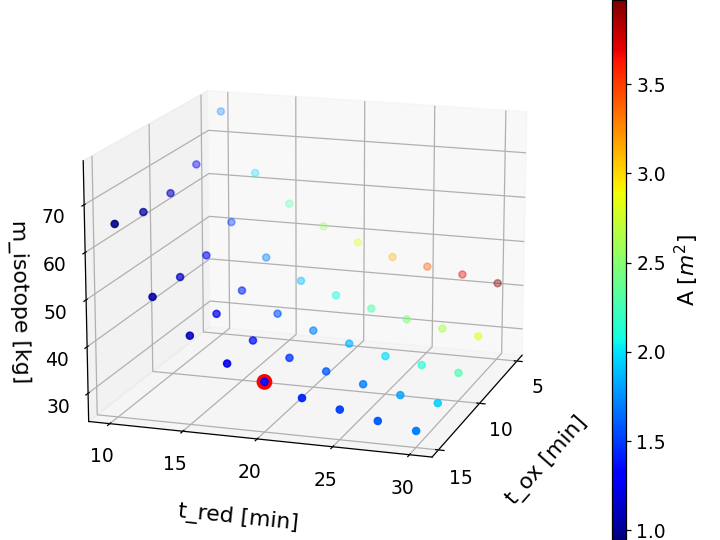

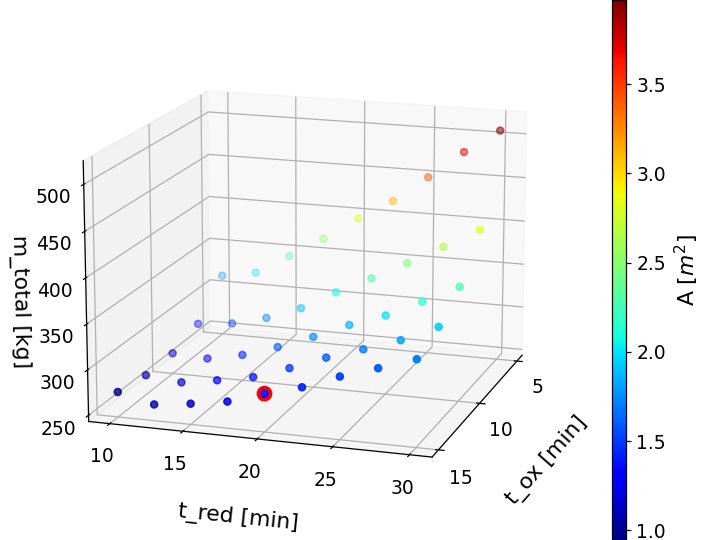


Figure 5: 3D EuNiO_3_-SrCO_3_ (100% ^90^Sr) calculation results over $t_{\mathrm{ox}}$ and $t_{\mathrm{red}}$; $T_{1}=300 ^{\circ}C$ , $T_{2}=475 ^{\circ}C$; (a) $m_{\mathrm{RI}}$ (b) $m_{\mathrm{total}}$

Table 2: EuNiO_3_-SrCO_3_ (100% ^90^Sr) calculation results: chosen case; $T_{1}=300 ^{\circ}C$ , $T_{2}=475 ^{\circ}C$

| $\boldsymbol{t}_{\mathbf{ox}}$ [min] | $\boldsymbol{t}_{\mathbf{red}}$ [min] | $\boldsymbol{m}_{\mathbf{AB}\mathbf{O}_{\mathbf{3}}}$ [kg] | $\boldsymbol{m}_{\mathbf{RI}}$  [kg] | $\boldsymbol{m}_{\mathbf{RIC}}$ [kg] | $\boldsymbol{m}_{\mathbf{cf}}$  [kg] | $\boldsymbol{m}_{\mathbf{total}}$[kg] | $\boldsymbol{V}_{\mathbf{comp}}$  [l] |
| --- | --- | --- | --- | --- | --- | --- | --- |
| 15.0 | 20.0 | 122.44 | 34.62 | 57.7 | 104.94 | 285.09 | 31.29 |
| ${\dot{\boldsymbol{V}}}_{\mathbf{ox}}$ [m^3^/s] | $\boldsymbol{P}_{\mathbf{blow}}$ [kW] | ${\dot{\boldsymbol{Q}}}_{\mathbf{ox}}$  [kW] | $\boldsymbol{\eta}_{\mathbf{util}}$ | $\boldsymbol{A}$  [m^2^] | $\boldsymbol{n}_{\mathbf{p}}$ | $\boldsymbol{d}_{\mathbf{comp}}$  [mm] | $\boldsymbol{\Delta T}_{\mathbf{CO}_{\boldsymbol{2}}}$  [K] |
| 73.13 | 3.66 | 73.64 | 0.05 | 1.32 | 38.0 | 0.72 | 61.34 |

$m_{{}^{90}\mathrm{Sr}(100\%)}$ of $34.62 kg correspond to a$thermal power ${(\dot{Q}}_{\mathrm{th}})$of$31.85 \mathrm{kW}$.

##

## EuNiO_3_-SrCO_3_ (60% ^90^Sr)


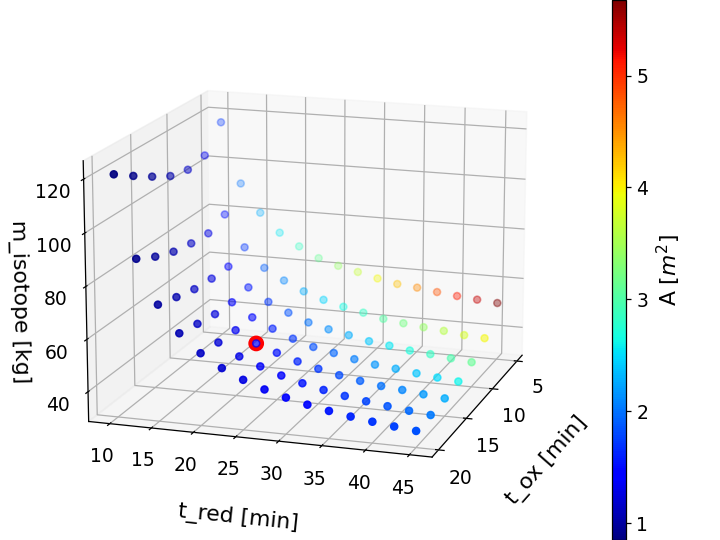

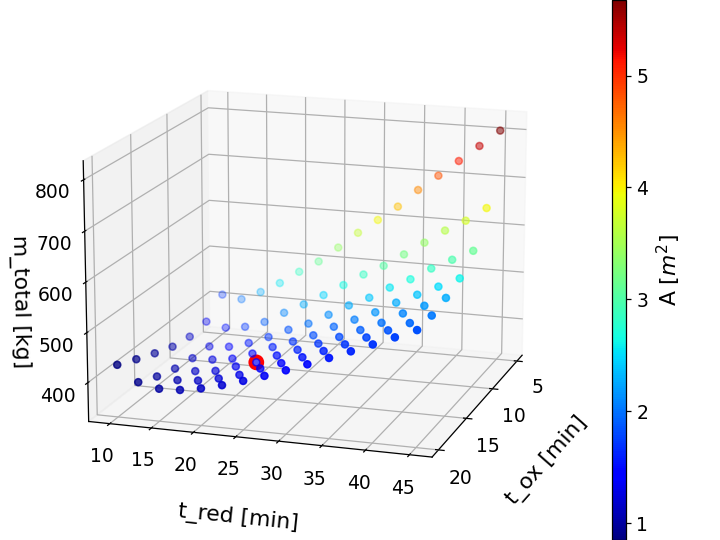


Figure 6: 3D EuNiO_3_-SrCO_3_ (60% ^90^Sr) calculation results over $t_{\mathrm{ox}}$ and $t_{\mathrm{red}}$; $T_{1}=300 ^{\circ}C$ , $T_{2}=425 ^{\circ}C$; (a) $m_{\mathrm{RI}}$ (b) $m_{\mathrm{total}}$

## EuNiO_3_-PuO_2_ (100% ^238^Pu)


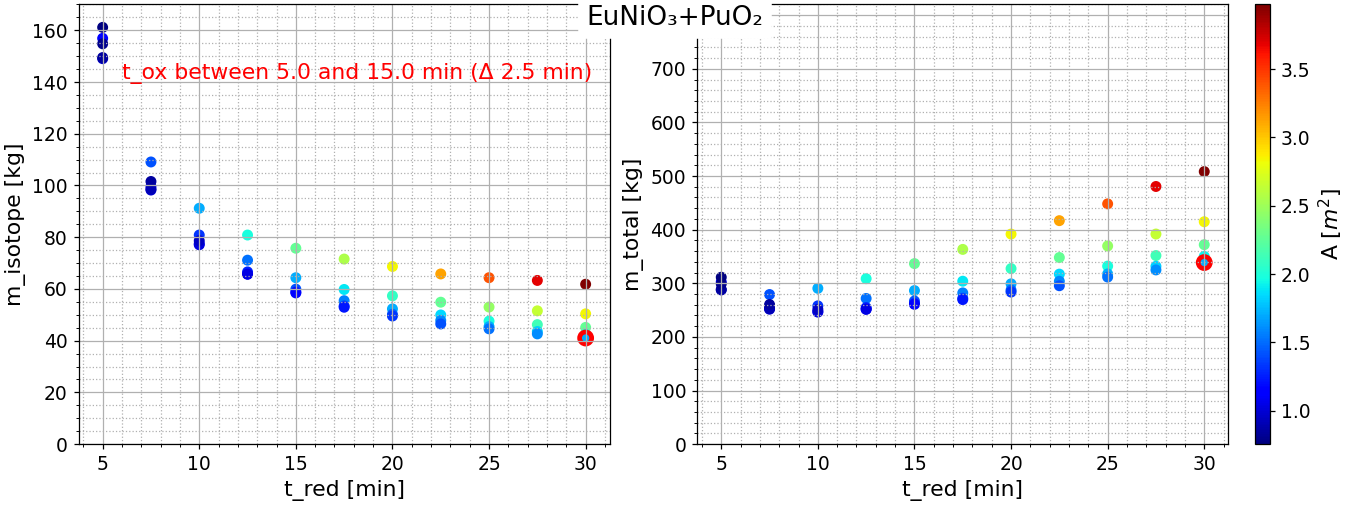


Figure 7: 2D EuNiO_3_-PuO_2_ (100% ^238^Pu) calculation results over $t_{\mathrm{ox}}$ and $t_{\mathrm{red}}$; $T_{1}=300 ^{\circ}C$ , $T_{2}=475 ^{\circ}C$; (a) $m_{\mathrm{RI}}$ (b) $m_{\mathrm{total}}$


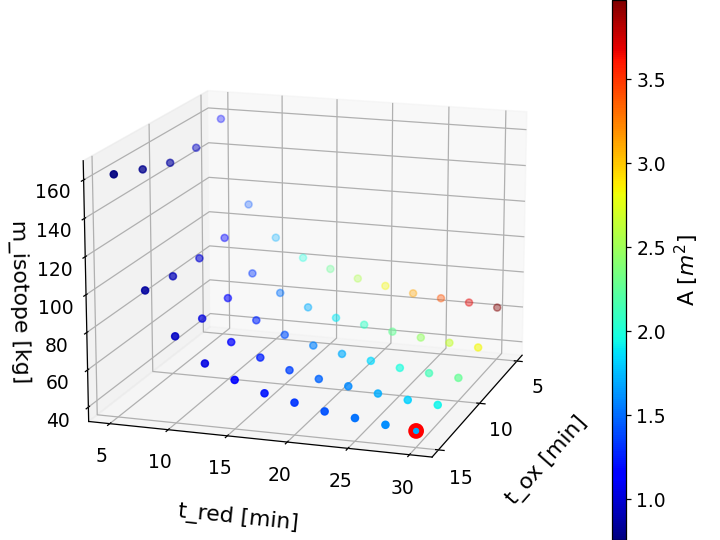

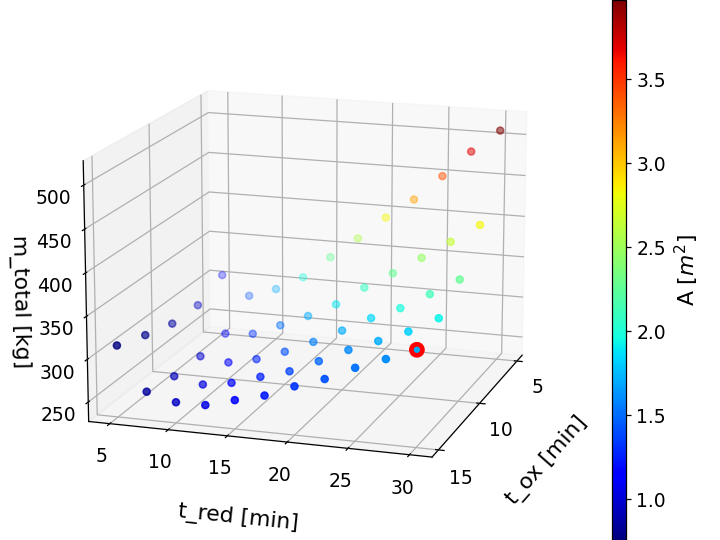


Figure 8: 3D EuNiO_3_-PuO_2_ (100% ^238^Pu) calculation results over $t_{\mathrm{ox}}$ and $t_{\mathrm{red}}$; $T_{1}=300 ^{\circ}C$ , $T_{2}=475 ^{\circ}C$; (a) $m_{\mathrm{RI}}$ (b) $m_{\mathrm{total}}$

Table 3: EuNiO_3_-PuO_2_ (100% ^238^Pu) calculation results: chosen case; $T_{1}=300 ^{\circ}C$ , $T_{2}=475 ^{\circ}C$

| $\boldsymbol{t}_{\mathbf{ox}}$ [min] | $\boldsymbol{t}_{\mathbf{red}}$ [min] | $\boldsymbol{m}_{\mathbf{AB}\mathbf{O}_{\mathbf{3}}}$ [kg] | $\boldsymbol{m}_{\mathbf{RI}}\boldsymbol{*}$  [kg] | $\boldsymbol{m}_{\mathbf{RIC}}$ [kg] | $\boldsymbol{m}_{\mathbf{cf}}$  [kg] | $\boldsymbol{m}_{\mathbf{total}}$[kg] | $\boldsymbol{V}_{\mathbf{comp}}$  [l] |
| --- | --- | --- | --- | --- | --- | --- | --- |
| 15.0 | 30.0 | 157.42 | 41.02 | 46.54 | 134.65 | 338.62 | 24.75 |
| ${\dot{\boldsymbol{V}}}_{\mathbf{ox}}$ [m^3^/s] | $\boldsymbol{P}_{\mathbf{blow}}$ [kW] | ${\dot{\boldsymbol{Q}}}_{\mathbf{ox}}$  [kW] | $\boldsymbol{\eta}_{\mathbf{util}}$ | $\boldsymbol{A}$  [m^2^] | $\boldsymbol{n}_{\mathbf{p}}$ | $\boldsymbol{d}_{\mathbf{comp}}$  [mm] | $\boldsymbol{\Delta T}_{\mathbf{CO}_{\boldsymbol{2}}}$  [K] |
| 94.03 | 4.7 | 69.28 | 0.07 | 1.7 | 43.0 | 0.44 | 44.88 |

${*m}_{{}^{238}\mathrm{Pu}(100\%)}$ of $41.02 \mathrm{kg}$ corresponds to a thermal power ($\dot{Q}_{\mathrm{th}})$of $23.38 \mathrm{kW}$.

## FOR 20% OXYGEN ABSORPTION $\boldsymbol{(}\boldsymbol{n}_{\mathbf{O}_{\mathbf{2, abs}}}\boldsymbol{=0.2}$)

## EuNiO_3_-Cm_2_O_3_ (95% ^244^Cm)


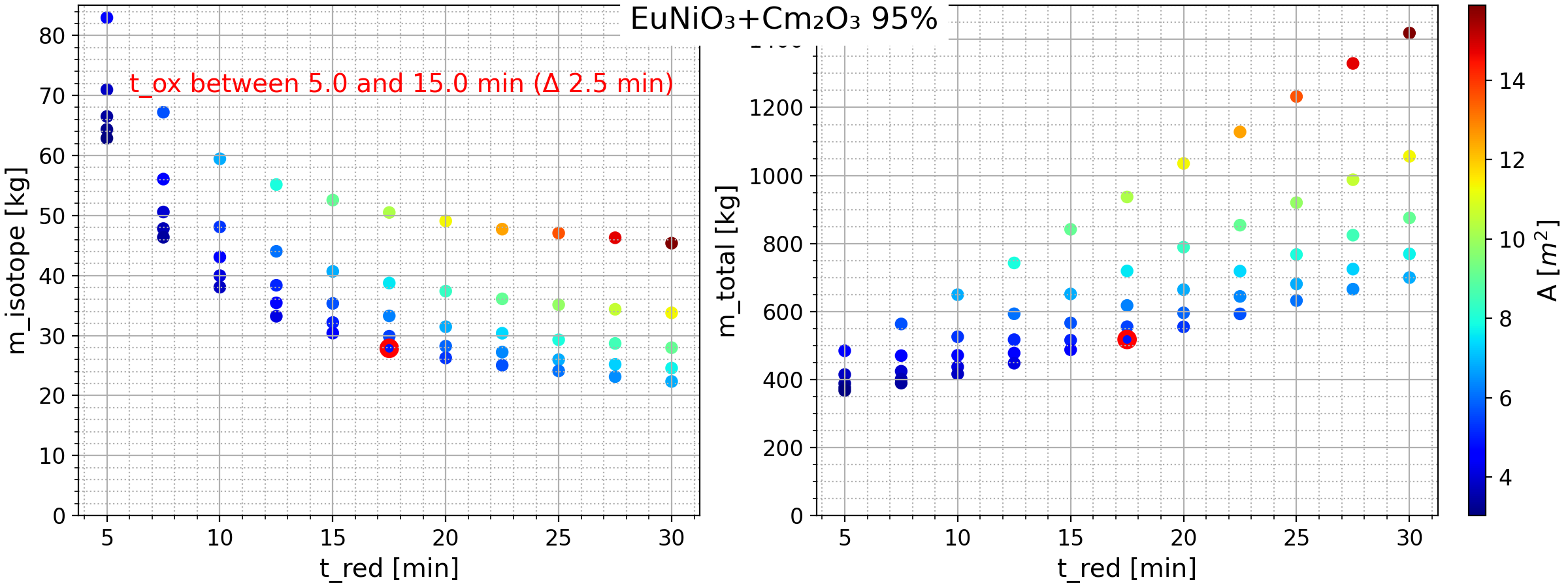


Figure 9: 2D EuNiO_3_-Cm_2_O_3_ (95% ^244^Cm) calculation results over $t_{\mathrm{ox}}$ and $t_{\mathrm{red}}$; $T_{1}=300 ^{\circ}C$ , $T_{2}=500 ^{\circ}C$; (a) $m_{\mathrm{RI}}$ (b) $m_{\mathrm{total}}$


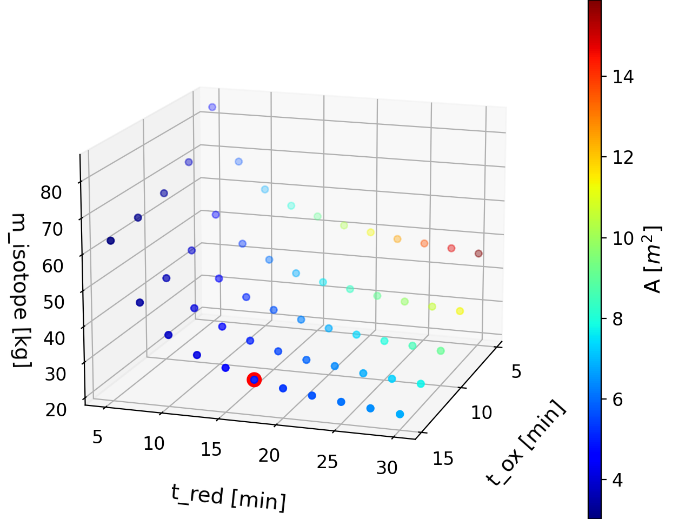

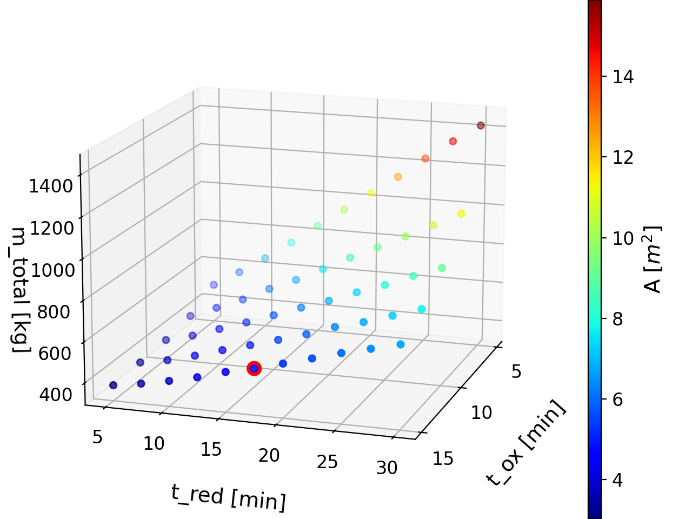


Figure 10: 3D EuNiO_3_-Cm_2_O_3_ (95% ^244^Cm) calculation results over $t_{\mathrm{ox}}$ and $t_{\mathrm{red}}$; $T_{1}=300 ^{\circ}C$ , $T_{2}=500 ^{\circ}C$; (a) $m_{\mathrm{RI}}$ (b) $m_{\mathrm{total}}$

Table 4: Chosen case EuNiO_3_-Cm_2_O_3_ (95% ^244^Cm) calculation results; $T_{\mathrm{ox}}=300 ^{\circ}C$ , $T_{\mathrm{red}}=500 ^{\circ}C$

| $\boldsymbol{t}_{\mathbf{ox}}$ [min] | $\boldsymbol{t}_{\mathbf{red}}$ [min] | $\boldsymbol{m}_{\mathbf{AB}\mathbf{O}_{\mathbf{3}}}$ [kg] | $\boldsymbol{m}_{\mathbf{RI}}\boldsymbol{*}$  [kg] | $\boldsymbol{m}_{\mathbf{RIC}}$ [kg] | $\boldsymbol{m}_{\mathbf{cf}}$  [kg] | $\boldsymbol{m}_{\mathbf{total}}$[kg] | $\boldsymbol{V}_{\mathbf{comp}}$  [l] |
| --- | --- | --- | --- | --- | --- | --- | --- |
| 15.0 | 17.5 | 98.66 | 27.86 | 30.6 | 388.54 | 517.80 | 15.62 |
| ${\dot{\boldsymbol{V}}}_{\mathbf{ox}}$  [m^3^/s] | $\boldsymbol{P}_{\mathbf{blow}}$ [kW] | ${\dot{\boldsymbol{Q}}}_{\mathbf{ox}}$  [kW] | $\boldsymbol{\eta}_{\mathbf{util}}$ | $\boldsymbol{A}$  [m^2^] | $\boldsymbol{n}_{\mathbf{p}}$ | $\boldsymbol{d}_{\mathbf{comp}}$  [mm] | $\boldsymbol{\Delta T}_{\mathbf{CO}_{\boldsymbol{2}}}$  [K] |
| 271.63 | 13.58 | 158.8 | 0.085 | 4.92 | 73.0 | 0.096 | 35.6 |
| $*m_{{}^{244}\mathrm{Cm}(95\%)}$of $27.86 \mathrm{kg}$ corresponds to a thermal power ${(\dot{Q}}_{\mathrm{th}})$ of $73.58 \mathrm{kW}$ | | | | | | | |

## EuNiO_3_-SrCO_3_ (100% ^90^Sr):


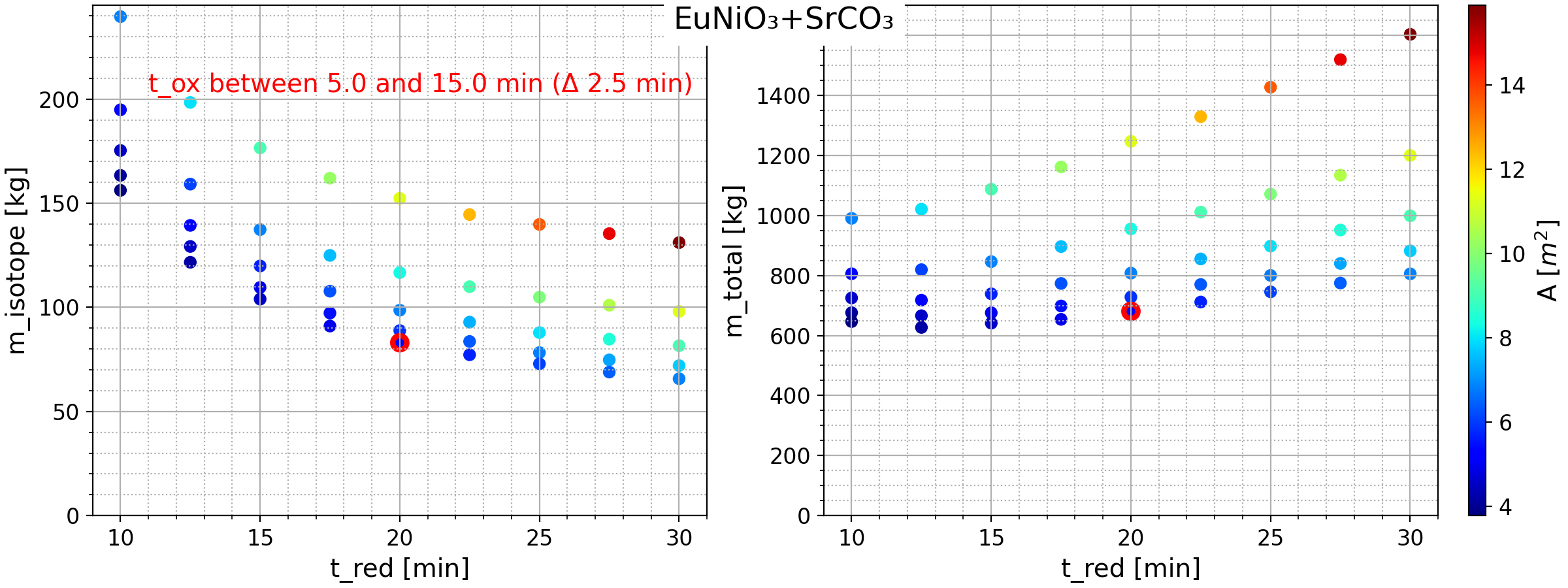


Figure 11: 2D EuNiO_3_-SrCO_3_ (100% ^90^Sr) calculation results over $t_{\mathrm{ox}}$ and $t_{\mathrm{red}}$; $T_{1}=300 ^{\circ}C$ , $T_{2}=475 ^{\circ}C$; (a) $m_{\mathrm{RI}}$ (b) $m_{\mathrm{total}}$


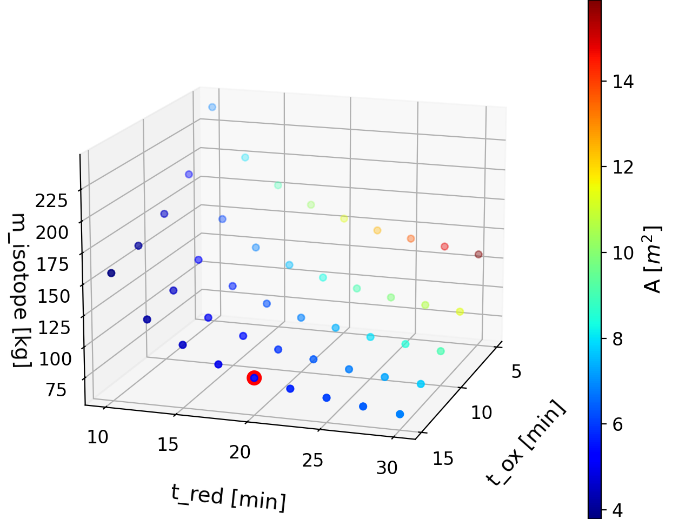

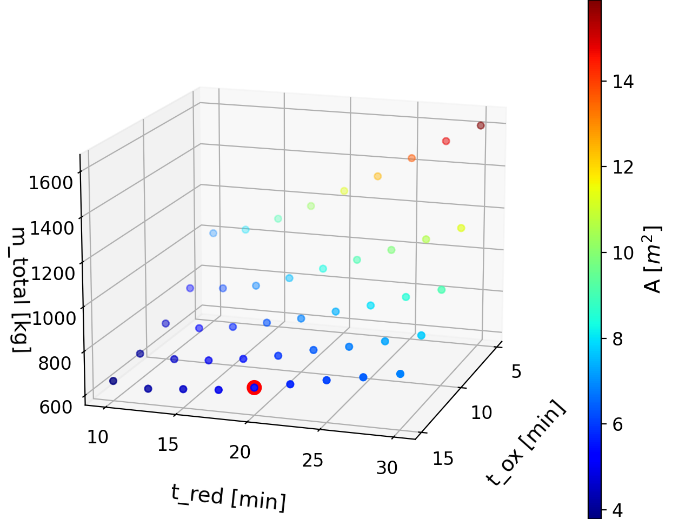


Figure 12: 3D EuNiO_3_-SrCO_3_ (100% ^90^Sr) calculation results over $t_{\mathrm{ox}}$ and $t_{\mathrm{red}}$; $T_{1}=300 ^{\circ}C$ , $T_{2}=475 ^{\circ}C$; (a) $m_{\mathrm{RI}}$ (b) $m_{\mathrm{total}}$

Table 5: EuNiO_3_-SrCO_3_ (100% ^90^Sr) calculation results: chosen case; $T_{1}=300 ^{\circ}C$ , $T_{2}=475 ^{\circ}C$

| $\boldsymbol{t}_{\mathbf{ox}}$ [min] | $\boldsymbol{t}_{\mathbf{red}}$ [min] | $\boldsymbol{m}_{\mathbf{AB}\mathbf{O}_{\mathbf{3}}}$ [kg] | $\boldsymbol{m}_{\mathbf{RI}}$  [kg] | $\boldsymbol{m}_{\mathbf{RIC}}$ [kg] | $\boldsymbol{m}_{\mathbf{cf}}$  [kg] | $\boldsymbol{m}_{\mathbf{total}}$[kg] | $\boldsymbol{V}_{\mathbf{comp}}$  [l] |
| --- | --- | --- | --- | --- | --- | --- | --- |
| 15.0 | 20.0 | 122.44 | 83.0 | 138.34 | 419.78 | 680.56 | 53.09 |
| ${\dot{\boldsymbol{V}}}_{\mathbf{ox}}$ [m^3^/s] | $\boldsymbol{P}_{\mathbf{blow}}$ [kW] | ${\dot{\boldsymbol{Q}}}_{\mathbf{ox}}$  [kW] | $\boldsymbol{\eta}_{\mathbf{util}}$ | $\boldsymbol{A}$  [m^2^] | $\boldsymbol{n}_{\mathbf{p}}$ | $\boldsymbol{d}_{\mathbf{comp}}$  [mm] | $\boldsymbol{\Delta T}_{\mathbf{CO}_{\boldsymbol{2}}}$  [K] |
| 292.53 | 14.63 | 177.51 | 0.08 | 5.3 | 76.0 | 0.30 | 36.96 |

$m_{{}^{90}\mathrm{Sr}(100\%)}$ of $83 kg correspond to a$thermal power ${(\dot{Q}}_{\mathrm{th}})$of$76.36 \mathrm{kW}$.

## EuNiO_3_-SrCO_3_ (60% ^90^Sr)


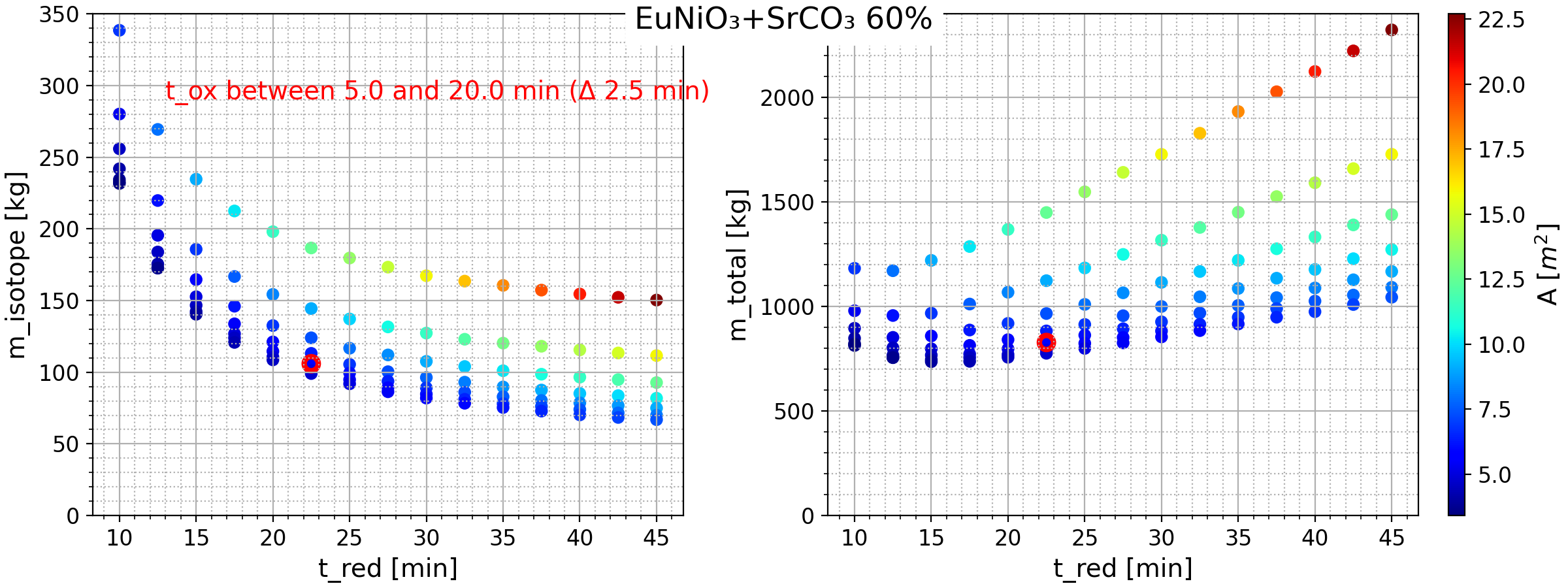


Figure 13: 2D EuNiO_3_-SrCO_3_ (60% ^90^Sr) calculation results over $t_{\mathrm{ox}}$ and $t_{\mathrm{red}}$; $T_{1}=300 ^{\circ}C$ , $T_{2}=425 ^{\circ}C$; (a) $m_{\mathrm{RI}}$ (b) $m_{\mathrm{total}}$


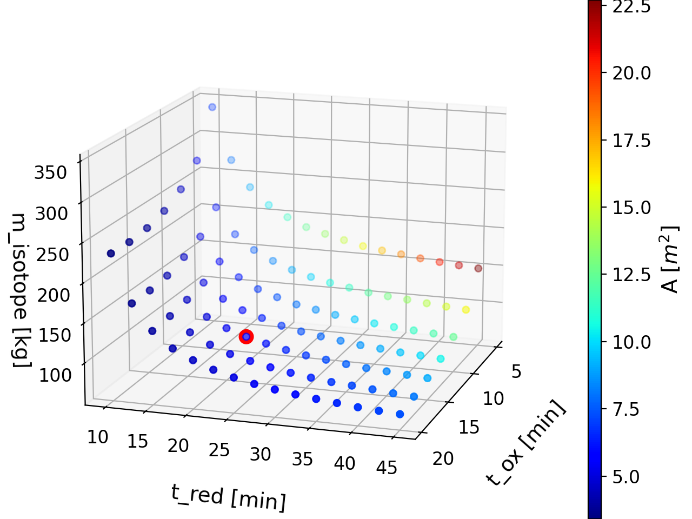

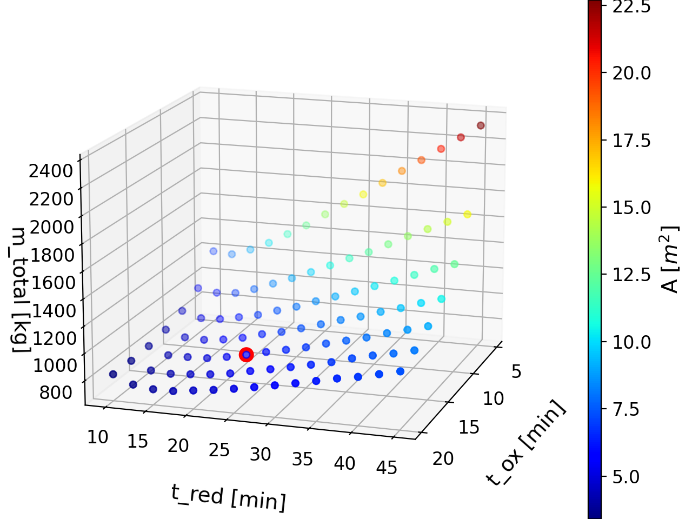


Figure 14: 3D EuNiO_3_-SrCO_3_ (60% ^90^Sr) calculation results over $t_{\mathrm{ox}}$ and $t_{\mathrm{red}}$; $T_{1}=300 ^{\circ}C$ , $T_{2}=425 ^{\circ}C$; (a) $m_{\mathrm{RI}}$ (b) $m_{\mathrm{total}}$

Table 6: Chosen case EuNiO_3_-SrCO_3_ (60% ^90^Sr) calculation results; $T_{\mathrm{ox}}=300 ^{\circ}C$ , $T_{\mathrm{red}}=425 ^{\circ}C$

| $\boldsymbol{t}_{\mathbf{ox}}$ [min] | $\boldsymbol{t}_{\mathbf{red}}$ [min] | $\boldsymbol{m}_{\mathbf{AB}\mathbf{O}_{\mathbf{3}}}$ [kg] | $\boldsymbol{m}_{\mathbf{RI}}\boldsymbol{*}$  [kg] | $\boldsymbol{m}_{\mathbf{RIC}}$ [kg] | $\boldsymbol{m}_{\mathbf{cf}}$  [kg] | $\boldsymbol{m}_{\mathbf{total}}$[kg] | $\boldsymbol{V}_{\mathbf{comp}}$  [l] |
| --- | --- | --- | --- | --- | --- | --- | --- |
| 15.0 | 22.5 | 199.84 | 105.9 | 176.56 | 451.66 | 828.1 | 73.34 |
| ${\dot{\boldsymbol{V}}}_{\mathbf{ox}}$ [m^3^/s] | $\boldsymbol{P}_{\mathbf{blow}}$ [kW] | ${\dot{\boldsymbol{Q}}}_{\mathbf{ox}}$  [kW] | $\boldsymbol{\eta}_{\mathbf{util}}$ | $\boldsymbol{A}$  [m^2^] | $\boldsymbol{n}_{\mathbf{p}}$ | $\boldsymbol{d}_{\mathbf{comp}}$  [mm] | $\boldsymbol{\Delta T}_{\mathbf{CO}_{\boldsymbol{2}}}$  [K] |
| 313.42 | 15.67 | 145.47 | 0.11 | 5.67 | 79.0 | 0.39 | 28.27 |
| ${*m}_{{}^{90}\mathrm{Sr}(60\%)}$ of $105.9 \mathrm{kg}$ corresponds to a thermal power $\dot{(Q}_{\mathrm{th}})$ of $58.46 \mathrm{kW}$. | | | | | | | |

## EuNiO_3_-PuO_2_ (100% ^238^Pu)


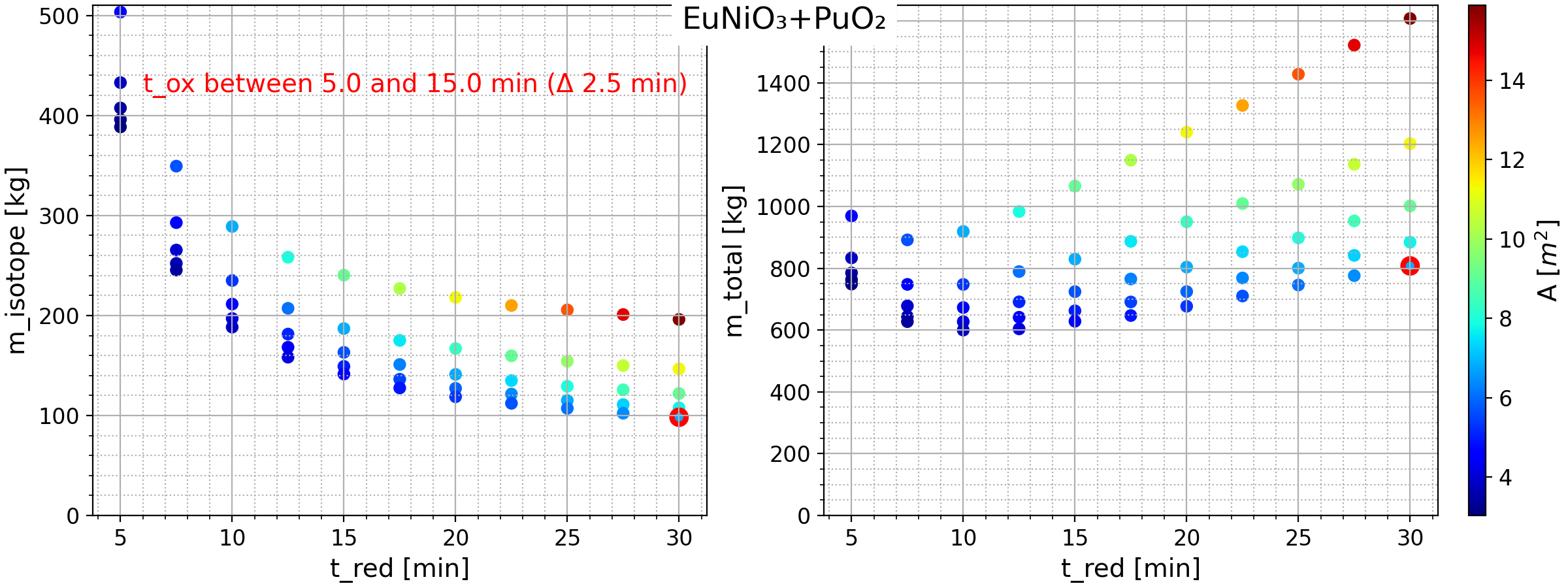


Figure 15: 3D EuNiO_3_-PuO_2_ (100% ^238^Pu) calculation results over $t_{\mathrm{ox}}$ and $t_{\mathrm{red}}$; $T_{1}=300 ^{\circ}C$ , $T_{2}=475 ^{\circ}C$; (a) $m_{\mathrm{RI}}$ (b) $m_{\mathrm{total}}$


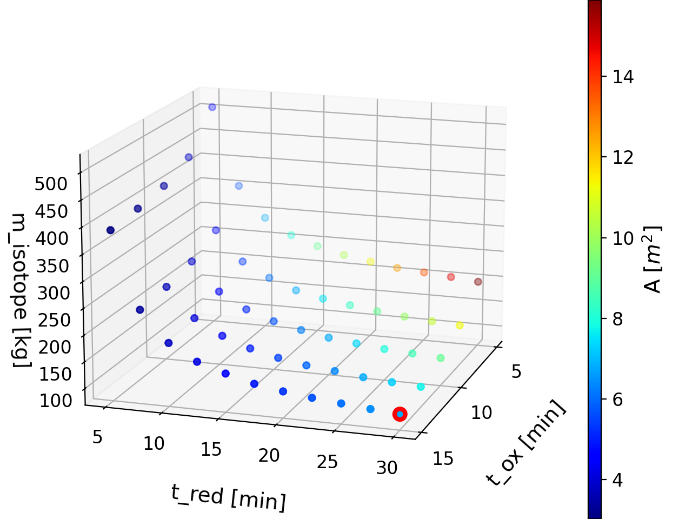

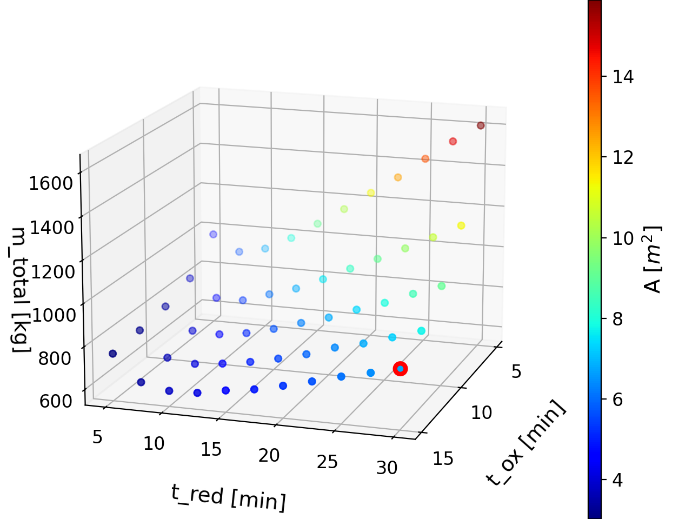


Figure 16: 3D EuNiO_3_-PuO_2_ (100% ^238^Pu) calculation results over $t_{\mathrm{ox}}$ and $t_{\mathrm{red}}$; $T_{1}=300 ^{\circ}C$ , $T_{2}=475 ^{\circ}C$; (a) $m_{\mathrm{RI}}$ (b) $m_{\mathrm{total}}$

Table 7: Chosen case EuNiO_3_-PuO_2_ (100% ^238^Pu) calculation results; $T_{\mathrm{ox}}=300 ^{\circ}C$ , $T_{\mathrm{red}}=475 ^{\circ}C$

| $\boldsymbol{t}_{\mathbf{ox}}$ [min] | $\boldsymbol{t}_{\mathbf{red}}$ [min] | $\boldsymbol{m}_{\mathbf{AB}\mathbf{O}_{\mathbf{3}}}$ [kg] | $\boldsymbol{m}_{\mathbf{RI}}\boldsymbol{*}$  [kg] | $\boldsymbol{m}_{\mathbf{RIC}}$ [kg] | $\boldsymbol{m}_{\mathbf{cf}}$  [kg] | $\boldsymbol{m}_{\mathbf{total}}$[kg] | $\boldsymbol{V}_{\mathbf{comp}}$  [l] |
| --- | --- | --- | --- | --- | --- | --- | --- |
| 15.0 | 30.0 | 157.42 | 98.3 | 111.5 | 538.6 | 807.55 | 31.12 |
| ${\dot{\boldsymbol{V}}}_{\mathbf{ox}}$ [m^3^/s] | $\boldsymbol{P}_{\mathbf{blow}}$ [kW] | ${\dot{\boldsymbol{Q}}}_{\mathbf{ox}}$  [kW] | $\boldsymbol{\eta}_{\mathbf{util}}$ | $\boldsymbol{A}$  [m^2^] | $\boldsymbol{n}_{\mathbf{p}}$ | $\boldsymbol{d}_{\mathbf{comp}}$  [mm] | $\boldsymbol{\Delta T}_{\mathbf{CO}_{\boldsymbol{2}}}$  [K] |
| 376.1 | 18.81 | 167.22 | 0.11 | 6.8 | 86.0 | 0.14 | 27.08 |

${*m}_{{}^{238}\mathrm{Pu}(100\%)}$ of $98.3 \mathrm{kg}$ corresponds to a thermal power ($\dot{Q}_{\mathrm{th}})$of $56.03 \mathrm{kW}$.

Table 8: Comparison of the most important calculation results for all investigated radioisotopes;
for selected balanced cases and optimized temperature swings for an oxygen uptake ratio of 20%, i.e. $n_{O_{2, \mathrm{abs}}}=0.2$.

|  |  | **Cm_2_O_3_ (95% ^244^Cm)** | **SrCO_3_ (100% ^90^Sr)** | **SrCO_3_ (60% ^90^Sr)** | **PuO_2_ (100% ^238^Pu)** |
| --- | --- | --- | --- | --- | --- |
| $\boldsymbol{A}$ | **[m^2^]** | 4.92 | 5.3 | 5.67 | 6.8 |
| $\boldsymbol{P}_{\mathbf{blow}}$ | **[kW]** | 13.58 | 14.63 | 15.67 | 18.81 |
| $\boldsymbol{m}_{\mathbf{RI}}$ | **[kg]** | 27.86 | 83.0 | 105.9 | 98.3 |
| ${\dot{\boldsymbol{Q}}}_{\mathbf{th}}$ | **[kW]** | 73.3 | 76.35 | 58.5 | 55.78 |
| $\boldsymbol{m}_{\mathbf{total}}$ | **[kg]** | 517.80 | 680.56 | 828.1 | 807.55 |

# Pre-Liquefaction Compression of Produced Oxygen

Index ‘0’ is the oxygen from the reactor chamber at reactor chamber pressure during reduction, but cooled down by contact with the Martian ambience.

$$T_{0}=273.15 K, \bar{p_{0}}=\bar{p_{O_{2},\mathrm{red}}}=0.005 \mathrm{bar}$$

Index ‘1’ is the compressed state to be fed to the liquefaction unit

$$p_{1}=1 \mathrm{bar}$$

Assuming an ideal gas, 100% O_2_, isentropic compression and 60% isentropic compression efficiency:

The isentropic index ${(n}_{O_{2}}=\kappa_{O_{2}}=\frac{c_{p}}{c_{v}}=1.35)$ was calculated from the O_2_ values at the Martian atmosphere conditions taken from ^17^. The compression power ${(P}_{t,01})$ is calculated as an hourly mean rather than the power demand during reduction.

$$P_{t,01}=\dot{m}_{O_{2}}\cdot y_{01}=\left( \frac{n_{O_{2}}}{n_{O_{2}}-1} \right)\cdot\frac{\dot{m}_{O_{2}}}{M_{O_{2}}}\cdot R\cdot T_{0}\cdot\left[ \left( \frac{p_{1}}{\bar{p_{0}}} \right)^{\frac{n_{O_{2}}-1}{n_{O_{2}}}}-1 \right]\cdot\frac{1}{\eta_{\mathrm{isen}}}$$

$$P_{t,01}=\left( \frac{1.35}{0.35} \right)\cdot\frac{2250\frac{g}{h}}{32\frac{g}{\mathrm{mol}}\cdot3600\frac{s}{h}}\cdot8.314\frac{J}{mol\cdot K}\cdot273.15 K\cdot\left[ \left( \frac{1 \mathrm{bar}}{0.005 \mathrm{bar}} \right)^{\frac{0.35}{1.35}}-1 \right]\cdot\frac{1}{0.6}=841 W$$

If the compression is divided into two steps (0.005 to 0.07 to 1 bar) and the flow is cooled to 0°C in between, this power can theoretically be reduced to the following value

$$P_{t,01,12}=2\cdot\left( \frac{1.35}{0.35} \right)\cdot\frac{2250\frac{g}{h}}{32\frac{g}{\mathrm{mol}}\cdot3600\frac{s}{h}}\cdot8.314\frac{J}{mol\cdot K}\cdot273.15 K\cdot\left[ \left( \frac{0.07 \mathrm{bar}}{0.005 \mathrm{bar}} \right)^{\frac{0.35}{1.35}}-1 \right]\cdot\frac{1}{0.6}=2\cdot283 W=566 W$$

# Reference

1. Josua Vieten. Perovskite Materials Design for Two-step Solar-thermochemical Redox Cycles. Unpublished, 2019.

2. Vieten, J. *et al.* Materials design of perovskite solid solutions for thermochemical applications. *Energy & Environmental Science* **12,** 1369–1384 (2019).

3. Riaz, A. *et al.* Redox Performance of Ceria–Vanadia Mixed-Phase Reticulated Porous Ceramics for Solar Thermochemical Syngas Production. *Energy & Fuels* (2021).

4. Rodat, S., Abanades, S., Boujjat, H. & Chuayboon, S. On the path toward day and night continuous solar high temperature thermochemical processes: A review. *Renewable and Sustainable Energy Reviews* **132,** 110061 (2020).

5. Fultz, B. Vibrational thermodynamics of materials. *Progress in Materials Science* **55,** 247–352; 10.1016/j.pmatsci.2009.05.002 (2010).

6. Halmann, M. & Steinfeld, A. Thermoneutral coproduction of calcium oxide and syngas by combined decomposition of calcium carbonate and partial oxidation/CO2-reforming of methane. *Energy & Fuels* **17,** 774–778 (2003).

7. Dolan, M. D., Beath, A. C., Hla, S. S., Way, J. D. & El Hawa, H. A. An experimental and techno-economic assessment of solar reforming for H_2_ production. *Int J Hydrogen Energ* **41,** 14583–14595 (2016).

8. Funken, K. Solar Energy Materials. *Solar Energy Materials* **24,** 370–385 (1991).

9. Fujimoto, S., Bilgen, E. & Ogura, H. Dynamic simulation of CaO/Ca(OH)_2 chemical heat pump systems. *Exergy, An International Journal* **2,** 6–14; 10.1016/s1164-0235(01)00035-8 (2002).

10. Graves, C., Ebbesen, S. D., Mogensen, M. & Lackner, K. S. Sustainable hydrocarbon fuels by recycling CO2 and H2O with renewable or nuclear energy. *Renewable and Sustainable Energy Reviews* **15,** 23-1; 10.1016/j.rser.2010.07.014 (2010).

11. Gao, Y., Jiang, J., Meng, Y., Yan, F. & Aihemaiti, A. A review of recent developments in hydrogen production via biogas dry reforming. *Energy Conversion and Management* **171,** 133–155 (2018).

12. Ambrosetti, G. & Good, P. A novel approach to high temperature solar receivers with an absorbing gas as heat transfer fluid and reduced radiative losses. *solar energy* **183,** 521–531 (2019).

13. Karasawa, H., Sasahira, A. & Hoshino, K. Thermal decomposition of SO 3. *International Journal of Nuclear Hydrogen Production and Applications,* 134–143 (2006).

14. Holladay, J. D., Hu, J., King, D. L. & Wang, Y. An overview of hydrogen production technologies. *Catalysis Today* **139,** 244–260; 10.1016/j.cattod.2008.08.039 (2009).

15. Rönsch, S. *et al.* Review on methanation–From fundamentals to current projects. *Fuel* **166,** 276–296 (2016).

16. Halmann, M. & Steinfeld, A. Hydrogen production and CO_2 fixation by flue-gas treatment using methane tri-reforming or coke/coal gasification combined with lime carbonation. *Int J Hydrogen Energ* **34,** 8061–8066; 10.1016/j.ijhydene.2009.08.031 (2009).

17. National Institute of Standards and Technology. Thermophysical Properties. Available at https://webbook.nist.gov/cgi/fluid.cgi?ID=C124389&Action=Page.
